# Transcriptome-wide mapping of milk somatic cells upon subclinical mastitis infection in dairy cattle

**DOI:** 10.1186/s40104-023-00890-9

**Published:** 2023-07-05

**Authors:** Vittoria Bisutti, Núria Mach, Diana Giannuzzi, Alice Vanzin, Emanuele Capra, Riccardo Negrini, Maria Elena Gelain, Alessio Cecchinato, Paolo Ajmone-Marsan, Sara Pegolo

**Affiliations:** 1 DAFNAE, University of Padova, Viale Dell’Università 16, Legnaro, PD 35020 Italy; 2 IHAP, Université de Toulouse, INRAE, ENVT, 23 Chemin Des Capelles, Toulouse, 31300 France; 3 IBBA, National Research Council, Via Einstein, Lodi, 26900 Italy; 4 DIANA, Università Cattolica del Sacro Cuore, Via E. Parmense 84, Piacenza, 29122 Italy; 5 BCA, University of Padova, Viale Dell’Università 16, Legnaro, PD 35020 Italy

**Keywords:** Data integration, Immune response, Milk somatic cells, RNA-sequencing, Subclinical mastitis

## Abstract

**Background:**

Subclinical intramammary infection (IMI) represents a significant problem in maintaining dairy cows’ health. Disease severity and extent depend on the interaction between the causative agent, environment, and host. To investigate the molecular mechanisms behind the host immune response, we used RNA-Seq for the milk somatic cells (SC) transcriptome profiling in healthy cows (*n* = 9), and cows naturally affected by subclinical IMI from *Prototheca* spp. (*n* = 11) and *Streptococcus agalactiae* (*S. agalactiae*; *n* = 11). Data Integration Analysis for Biomarker discovery using Latent Components (DIABLO) was used to integrate transcriptomic data and host phenotypic traits related to milk composition, SC composition, and udder health to identify hub variables for subclinical IMI detection.

**Results:**

A total of 1,682 and 2,427 differentially expressed genes (DEGs) were identified when comparing *Prototheca* spp. and *S. agalactiae* to healthy animals, respectively. Pathogen-specific pathway analyses evidenced that *Prototheca*’s infection upregulated antigen processing and lymphocyte proliferation pathways while *S. agalactiae* induced a reduction of energy-related pathways like the tricarboxylic acid cycle, and carbohydrate and lipid metabolism. The integrative analysis of commonly shared DEGs between the two pathogens (*n* = 681) referred to the core-mastitis response genes, and phenotypic data evidenced a strong covariation between those genes and the flow cytometry immune cells (*r*^2^ = 0.72), followed by the udder health (*r*^2^ = 0.64) and milk quality parameters (*r*^2^ = 0.64). Variables with *r* ≥ 0.90 were used to build a network in which the top 20 hub variables were identified with the Cytoscape cytohubba plug-in. The genes in common between DIABLO and cytohubba (*n* = 10) were submitted to a ROC analysis which showed they had excellent predictive performances in terms of discriminating healthy and mastitis-affected animals (sensitivity > 0.89, specificity > 0.81, accuracy > 0.87, and precision > 0.69). Among these genes, *CIITA* could play a key role in regulating the animals’ response to subclinical IMI.

**Conclusions:**

Despite some differences in the enriched pathways, the two mastitis-causing pathogens seemed to induce a shared host immune-transcriptomic response. The hub variables identified with the integrative approach might be included in screening and diagnostic tools for subclinical IMI detection.

**Supplementary Information:**

The online version contains supplementary material available at 10.1186/s40104-023-00890-9.

## Background

Mastitis in dairy cattle is a well-established problem that firstly affects animal welfare but also hinders milk production and quality, leading to significant economic losses at the expense of farmers [1]. Mastitis typically occurs in response to the penetration of a wide range of microorganisms in the mammary gland. It exists in two forms: the clinical one, with overt signs of inflammation, udder swelling, and changes in milk physical composition, and the subclinical one, where usually the only alteration observed is the increase in the somatic cell count (SCC) derived from the proliferation and migration of the immune cells in the udder [2]. Subclinical mastitis is the most challenging form, estimated to be 15–40 times more frequent than its clinical counterpart [3]. It represents a significant source of infection for the animals within the herds leading to an essential decrease in milk production [4] while going unnoticed. The most common causative agents of mastitis, accounting for over 80% of the infections [5], are *Escherichia coli* (*E. coli*), *Staphylococcus aureus* (*S. aureus*), *Streptococcus agalactiae *(*S. agalactiae*), *Streptococcus uberis* (*S. uberis*) and *Streptococcus dysgalactiae* (*S. dysgalactiae*)*.* However, in the last few decades, the spreading of other microorganisms, like microalgae of the genus *Prototheca,* has rapidly become an emerging threat for the dairy sector, mainly because nowadays, there is still no treatment effective towards this type of microorganism [6].

The different etiology of invading pathogens can trigger a diverse host immune response, consequently affecting the extent and outcome of the infection [7]. For example, Gram-negative bacteria like *E. coli* usually induce an intense stimulation of cytokine production, leading to fully activating both the local and generalized host-immune response [8, 9], Gram-positive pathogens elicit a weaker immune reaction with usually no systemic repercussion [10]. In addition, the immuno-cytofluorimetric study conducted in subclinical mastitis induced naturally by *S. agalactiae* and *Prototheca* [11] highlighted a differential immune reaction between the two microorganisms, primarily directed towards an innate response in the case of *S. agalactiae*, as opposed to the adaptive response triggered by *Prototheca* spp. Therefore, a deeper understanding of the pathogen-specific molecular mechanisms underlying the pathogenesis of mastitis and the induced immune response is pivotal for uncovering new ways of predicting the infection outcome and designing practical diagnostic and therapeutic tools for battling this costly disease.

In this context, RNA sequencing (RNA-Seq) represents a suitable tool for investigating the complexity of the host–pathogen interaction [12]. Different transcriptomic studies have evaluated the mammary gland [10, 12] or hepatic [13] response to intra-mammary infection with different types of pathogens in cows. However, only a few studies evaluated the changes in somatic milk cells (SC) transcriptome [14, 15] in response to subclinical intra-mammary infection (IMI), and, more importantly, to the best of our knowledge, no studies are currently available on the investigation of milk SC transcriptomic signature of *Prototheca* spp. infection in dairy cattle. Finally, most of the previous transcriptomic studies were conducted using experimentally induced models of clinical mastitis [16], while less information is available on naturally occurring subclinical mastitis [17].

Work on identifying putative candidate genes associated with mastitis has already been carried out in genome-wide association studies (GWAS) and transcriptomic profiling [14, 18, 19]. However, the concordance among these studies could be higher, highlighting the difficulty in identifying reliable and reproducible biomarkers for mastitis detection and mastitis resistance. In this context, integrating transcriptomic with phenomic information might represent the ultimate step not only to strengthen the information on the complexity of the molecular system by reinforcing complementary levels of knowledge but also to create more reliable prediction models [20].

Therefore, this study aimed to i) evaluate the milk SC transcriptomic signatures upon natural infection of *S. agalactiae* and *Prototheca* spp., ii) integrate transcriptomic and phenomic information to explain better the complexity underlying the molecular mechanisms of mastitis, and identify hub variables for early mastitis detection and prediction and, iii) perform a meta-analysis using three publicly available datasets to confirm the reproducibility of our results.

## Methods

### Animal cohort, housing, and diet

Thirty-one multiparous Holstein cows (ranging from 3 to 7 years of age) between 98 and 448 days in milk (DIM) were selected from a commercial herd of 450 lactating cows (Veneto region, Italy) regularly monitored for *S. agalactiae* and *Prototheca* spp. between January and February 2021. Herd selection was based firstly on a prevalence study conducted by the *Istituto Zooprofilattico delle Venezie* (IZSVe) for the identification of the most common pathogen responsible for mastitis in the Veneto region and on ease of access to the farm location and the cooperation of the dairy farm owners and their associated veterinary practices. For successful participation in the study, we required the following criteria: (i) absence of clinical signs of infection; (ii) no antibiotic treatment or anti-inflammatory medications before enrollment; (iii) being multiparous and non-pregnant; and (iv) having > 98 DIM. Moreover, we required that animals used as negative control had no previous history of mastitis. Information was collected from the herd management software (Dairy Comp Sata, Alta Italia Srl, Milan, Italy). Based on these criteria, an initial bacteriological screening (time 0, T0) was performed on 188 lactating cows to identify healthy individuals and cows with subclinical mastitis from *S. agalactiae* or *Prototheca* spp. Animals with co-infection were excluded from the experiment. Moreover, cows with chronic mastitis cases (apparently healthy cows with lumps palpable in the udder and milk quality changes) were not enrolled. Following the bacteriological test results, we created three experimental groups from eligible animals: (i) healthy individuals (*n* = 9) with a negative bacteriological examination in all glands at T0 and time 1 (T1, two weeks after T0); (ii) naturally infected animals with *S. agalactiae* (*n* = 11) and (iii) naturally infected animals with *Prototheca* spp. *(n* = 11*)*. At T1, a second bacteriological assessment was made on all the animals enrolled to confirm the bacteriological evaluation made at T0 (Table S1).

Cows were fed a total mixed ration formulated to meet or exceed the requirements of mid-lactation dairy cattle, mainly based on corn silage, sorghum silage, and concentrate. Feed was delivered once a day at 8:00, and the amount fed was adjusted daily to allow for a minimum of 5% refusals. Drinking water was available in automatic water bowls, and cows were milked twice daily, from 2:00 to 6:00, and from 14:00 to 18:00. Individual cow milk yield was recorded at each milking using herd software.

Animal health was managed by the farmers and local veterinarians, who intervened when needed. All cows were subjected to the same management practices and environment to ensure sample homogeneity.

### Ethical statement

This study was part of the LATSAN project that aimed to develop innovative tools for evaluating and studying mammary gland health and improving dairy cows’ nutritional milk quality and coagulation properties. The research was approved by the Ethical Animal Care and Use Committee (OPBA—Organismo Preposto al Benessere degli Animali) of the Università Cattolica del Sacro Cuore and by the Italian Ministry of Health (protocol number 510/2019-PR of 19/07/2019).

### Milk sample collection

Before morning milking, ~ 150 mL of milk from all quarters (pool sample) was aseptically collected from each animal according to the National Mastitis Guidelines [21]. Briefly, teat ends were externally cleaned with commercial pre-milking disinfectant, dried with individual towels, and then washed again with alcohol 70%. Composite milk of the four glands was then collected after discarding the first streams of foremilk from each quarter and stored at 4 °C before microbiological analysis. Four milk aliquots (~ 50 mL) of each milk sample were collected and gently mixed separately into sterile tubes for analysis as follows: (i) microbiological analysis; (ii) evaluation of milk composition, SCC, and differential somatic cell count (DSCC) measurement; (iii) milk flow cytometry analysis; and (iv) RNA extraction and transcriptomic analysis. All the samples were immediately refrigerated at 4 °C to minimize the metabolic activity of cells and enzymes and keep the bacteriological composition as stable as possible. Samples were transported under refrigerated conditions (4 °C) to the different laboratories.

### Microbiological analysis

Microbiological examination of milk samples was conducted at the IZSVe laboratories (Legnaro, PD, Italy). After reception (within 4 h after sample collection), samples were frozen and analyzed within 3 d. Pegolo et al. [11] reported specifics of the microbiological analyses in detail. Briefly, 10 μL of every composite sample were inoculated in each of the following selective media: i) Baird Parker agar with rabbit plasma fibrinogen (BP-RPF; Biokar Diagnostics, Beauvais, France), ii) tallium kristalviolette tossin agar (TKT; IZSVe internal production), and iii) *Prothoteca* isolation medium (PIM; IZSVe internal production). Suspected colonies of *S. agalactiae* were confirmed using the Christie–Atkins–Munch-Peterson test [21] after 24 h of incubation. At the same time, *Prototheca* isolation medium plates were observed at 24, 48 and 72 h, and the wet mount method confirmed suspected colonies [21].

### Milk composition and quality traits

Milk composition (protein, casein, lactose, fat, and urea content), milk conductivity (mS/cm), and milk pH analysis were carried out on fresh samples using an FT6000 Milkoscan infrared analyzer (Foss A/S, Hillerød, Denmark). SCC and DSCC were measured through the Fossomatic 7 DC analyzer (Foss A/S).

### Flow cytometry analysis

A 50-mL aliquot of each sample was immediately processed in the Comparative Biomedicine Department (BCA) cell laboratory of the University of Padova (Italy) for flow cytometry analysis. In all cases, analyses were performed within 12 h after sample collection with milk stored at 4 ºC. The whole flow cytometry methodology and analysis are reported in the work of Pegolo et al. [11].

Briefly, for each sample, flow cytometry analysis was run in four tubes containing: 1) only cells (no antibodies; used as a negative control); 2) cd4pe-cd8alexa fluor 647; 3) cd11bfitc-cd14pe; and 4) cd45fitc-cd21pe-cd18alexa fluor 647. Flow cytometric analyses were performed using a CyFlow Space flow cytometer (Sysmex Partec GmbH, Norderstedt, Germany) fitted with a blue laser (488 nm), a red laser (635 nm) and a UV laser. The data were analyzed with the FlowMax software version 2.82 (Sysmex Partec GmbH, Norderstedt, Germany). The morphology and complexity of the cells were evaluated in an FSC vs. SSC dot plot; total white blood cells were identified as CD45 and CD18 positive events; polymorphonuclear cells as CD11b positive CD14 negative events; macrophages as CD11b and CD14 positive events; T-helper lymphocytes as CD4 positive and CD8 negative events; T cytotoxic lymphocytes as CD8 positive and CD4 negative events; B lymphocytes as CD45, CD21, and CD18 positive events. In this study, we considered for the statistical analyses only animals subjected to RNA-seq analyses which are part of the broader cohort of animals previously analyzed [11].

The Kruskal Wallis and Dunn test assessed significance among the experimental groups for pairwise comparison of milk production, composition, and flow cytometry variables. The significance was set at *P* < 0.05.

### RNA extraction from milk somatic cells

A 50-mL aliquot from each sample was first centrifuged at 2,000 × *g* for 10 min at 4 °C. The fat layer and the supernatant were discarded, and the cell pellet was then washed with 50 mL of PBS with ethylenediaminetetraacetic acid (EDTA) at 0.05 mmol/L, pH 7.2. Samples were then re-centrifuged at 1,500 × *g* for 10 min at 4 °C, the supernatant discarded, and the pellet was re-suspended in 800 μL of Trizol (Invitrogen, Carlsbad, CA, USA) and stored at −80 °C until the RNA extraction.

Total RNA was extracted from the Trizol reagent and purified using a NucleoSPin miRNA kit (Macherey–Nagel, Düren, Germany), following the combined protocol with TRIzol lysis with small and large RNA in one fraction (total RNA). RNA concentration and quality were determined by Agilent 2100 Bioanalyzer (Santa Clara, CA, USA). Extracted RNA was stored at −80 °C until use.

### Library preparation

The 31 RNA samples, including control (*n* = 9), positive for *S. agalactiae* (*n* = 11), and positive for *Prototheca* (*n* = 11), were subsequently sent on dry ice to the Nuova Genetica Italiana (NGI, Como, Italy) facility for library preparation and sequencing. MGIEasy rRNA Depletion kit V1.1 (MGI Tech Co., Ltd., Shenzen, China) was used to remove ribosomal rRNA and maximise unique sequencing reads. RNA-seq libraries were then prepared from 500 ng of total RNA using the MGIEasy RNA Library Prep Set V3.1 (MGI Tech Co., Ltd., Shenzen, China), according to the manufacturer’s protocol. RNA-seq experiments were performed on a DNBSEQ-G400 high throughput machine (MGI Tech CO., Ltd.) using a paired-end approach using the DNBSEQ-G400 sequencing kit (MGI TechCo., Ltd., Shenzen, China).

### RNA-seq data processing and analysis

Data pre-processing was made following the consensus pipeline built by Overbey et al. [22]. First, the quality control of RNA sequences was assessed with the FastQC software (v. 0.11.9). Clean reads were obtained by removing low-quality bases and adaptors with the TrimGalore software (v. 0.6.4) [23]. FastQC was used again on the trimmed sequences to check the quality of the reads. MultiQC package (v.1.8) [24] was run to create summary statistics reports that included the sample quality control result categories from FastQC across all experiment samples. The sample information of clean data is shown in Table S2.

The paired-end clean reads were aligned against the *Bos taurus* DNA reference genome (ARS-UCD1.2) from the USDA’s Agricultural Research Service with the splice-aware STAR (v.2.7.3a) [25]. The genome indexing was performed using ARS-UCD1.2 as the reference FASTA and the Ensembl gene annotation file (Bos_taurus.ARS-UCD1.2.106.gtf.gz; http://ftp.ensembl.org/pub/release-106/gtf/bos_taurus/).

We subsequently used RSEM (v.1.3.3) [26] to quantify gene expression. Similar to STAR, RSEM was run in two distinct phases. The first phase used the reference genome and GTF files to prepare indexed genome files. The second phase used the indexed files and the mapped reads from STAR to assign counts to each gene.

### Gene expression evaluation and differential expression analysis

Counts filtering, data normalization, and differential expression analysis were performed in R studio (R v.4.1.2, R studio v. 1.4.1103). Only protein-coding genes were considered for the analysis.

We first normalized the transcriptome count matrix with the sequencing depth for each sample by calculating counts per million (CPM). We filtered out genes expressed in less than 10 samples with CPM < 0.5 using the edgeR package (v. 3.36.0) [27]. Genes failing these criteria were removed before the exploration and differential expression analysis.

Exploratory analysis of the expressed genes matrix was performed using unsupervised principal component analysis (PCA) and the multidimensional scaling (MDS) analysis after the regularized-logarithm transformation (edgeR) or variance stabilizing transformation in DESeq2 (v. 1.34.0) [28].

Differentially expressed gene (DEG) analysis was performed pairwise using DESeq2 and edgeR packages: i) negative animals vs. *S. agalactiae* naturally infected animals; ii) uninfected subjects vs. *Prototheca* infected animals; and iii) *S. agalactiae* vs. *Prototheca* infected individuals. Then, the *voom()* function from the limma R package (v.3.50.0) was used to fit a generalized linear regression model to correct the data with the group as a fixed effect. The group factor and the cow dependency were included in the generalized linear model using the *nbinomWaldTest()* function, which estimates and tests the significance of regression coefficients with the following explicit parameter settings: betaPrior = FALSE, maxit = 5,000, useOptim = TRUE, useT = FALSE, useQR = TRUE, minmu = 0.5. The *P*-values were adjusted for multiple testing using the Benjamini and Hochberg procedure (FDR, false discovery rate).

Only DEGs with an adjusted *P-*value < 0.05 and shared between DESeq2 and edgeR approaches were used for the downstream pathway analysis.

### Functional pathway analysis

The shared list of DEGs for each comparison was fed to the Cytoscape (v. 3.9.1, http://cytoscape.org) ClueGo plugin (v. 2.5.8) [29] software to identify relevant biological processes and immune systems networks. A minimum of 10 genes were needed to be associated with a term. These genes would represent at least 4% of the total number of related genes. Only pathways with a *P-*value < 0.05 (Bonferroni step-down correction) were retained. Results were illustrated as a functionally grouped network of terms, having the most significant one as a leading term. The edges that show term-to-term interactions were obtained using a Kappa score of 0.4.

Then, coupled with DEG-driven approaches, we used the pathifier algorithm from the pathifier R package (v.1.32.0) [30], which by transforming the whole transcriptome expression data into pathway-level information, infers the pathway deregulation scores by measuring how much the gene expression of a sample deviates from normal behavior. Kyoto Encyclopedia of Genes and Genomes (KEGG) annotation was used, and the *quantify_pathway_deregulation()* function was used to quantify the deregulation scores. Euclidean distance was used to calculate samples’ distances, then visualized using the Ward.D2 clustering method in a heatmap.

### Phenomics: complementary data integration approaches

A global non-metric multidimensional scaling (NMDS) ordination was used to extract, visualize and summarize the variation in the transcriptome (the “response variable”) using the vegan R package (v.2.5.7) [31]. Stress values were calculated to determine the number of dimensions for each NMDS. Stress values measure how much the distances in the reduced ordination space depart from those in the original* p*-dimensional space. High-stress values indicate a greater possibility that structuring observations in the ordination space is entirely unrelated to the actual full-dimensional space. Then, the explanatory variables related to milk composition and quality traits, cytometry cell profiles, and host morphometric parameters were fitted to the ordination plots using the *envfit()* function in the vegan R package [32] with 10,000 permutations. The *envfit()* function performs multivariate analysis of variance (MANOVA) and linear correlations for categorical and continuous variables. The effect size and significance of each covariate were determined by comparing the difference in the centroids of each group relative to the total variation, and all *P*-values derived from the *envfit()* function were Benjamini–Hochberg adjusted. The obtained *r*^2^ gives the proportion of variability (that is, the main dimensions of the ordination) that can be attributed to the explanatory variables.

As a second integrative approach, the *N*-integration algorithm DIABLO (Data Integration Analysis for Biomarker discovery using Latent Components) of the mixOmics R package [33] (http://mixomics.org/, v. 6.18.1) was used. We combined host-centered transcriptomics with phenomics data to achieve this integrated perspective, coined holo-omics [34]. It is to be noted that, in the case of the *N-*integration algorithm DIABLO, the variables of all the data sets were also centered and scaled to unit variance before integration. In this case, the relationships among all data sets were studied by adding a different categorical variable, e.g.*,* the infection status of cows. Healthy cows (*n* = 9) were compared to infected individuals (*n* = 22). DIABLO seeks to estimate latent components by modeling and maximizing the correlation between pairs of pre-specified datasets to unravel similar functional relationships [35]. The model was first fine-tuned using leave-one-out cross-validation by splitting the data into training and testing. Then, classification error rates were calculated using balanced error rates (BERs) between the predicted latent variables and the class labels' centroid. Only interactions with |*r*| ≥ 0.80 were visualized using CIRCOS.

### Identification and validation of hub variables

To visualize the high-confidence variable co-associations, only those with |*r*| ≥ 0.90 and more than 15 connections were automatically visualized using the organic layout algorithm in Cytoscape (version 3.9.1). The Molecular Complex Detection (MCODE) Cytoscape plug-in (version 2.01, [36]) was adopted to detect densely connected modules within the interaction network. MCODE scores ≥ 3 were set as a cut-off criterion with default parameters.

Finally, cytoHubba (version 0.1) [37], a Cytoscape plug-in, was used to explore the network modules for identifying hub genes, defined as genes having high correlation in candidate modules. The top 20 variables were identified and ranked using the Maximal Clique Centrality (MCC) method.

To validate the abovementioned hub genes as putative markers for mastitis infection, we performed the receiver operating characteristics (ROC) and precision-recall analyses using the R package pROC (v. 1.18.0) package to quantify the infection status predictive power of hallmark variables.

### The meta-analysis cohort

To confirm the reproducibility of our prediction results in healthy and infected individuals, studies on the transcriptome of the milk somatic cell in dairy cows with high-throughput RNA sequence data in.fastq format deposited in publicly accessible databases and available metadata were retrieved.

We obtained 81 somatic cell transcriptomic samples from three independently published studies as an orthogonal dataset. The three studies were labeled as Seo [38], Asselstine [14], and Niedziela [15]. Data included acute and subclinical infection regarding both naturally and experimentally infected animals. Raw sequence data and metadata from the Seo study were available at GSE60575 in the GEO database. In contrast, Asselstine study raw fastq files and metadata were retrieved from the NCBI under PRJNA544129 Bioproject accession number. Raw fastq files and metadata of the Niedziela study were available in the European Nucleotide Archive (ENA) repository with the project number PRJEB43443. The published data was pre-processed and annotated as described above. In this validation set, “healthy” subjects were defined as those reported as not being infected in the original research; alternatively, “mastitis” subjects were defined as those diagnosed with mastitis infection either by the California Mastitis Test [14] or after 24 h from the disease onset with two different strains of *Staphylococcus aureus* [15].

Ten-fold cross-validation sparse Partial Least Squares Discriminant Analysis (sPLS-DA) was employed to evaluate the prediction model’s performance and validate the essential genes responsible for the differences between groups using the mixOmics R package. The DESeq2 R package quantified differences between groups' relative gene abundance.

## Results

### Animals and data

The 31 Holstein cows enrolled in this study were, on average, 4 years of age. The mean DIM at enrollment for all dairy cattle was 235 d, and the mean parity was 2.5, ranging from 2 to 5.

**Fig. 1:**
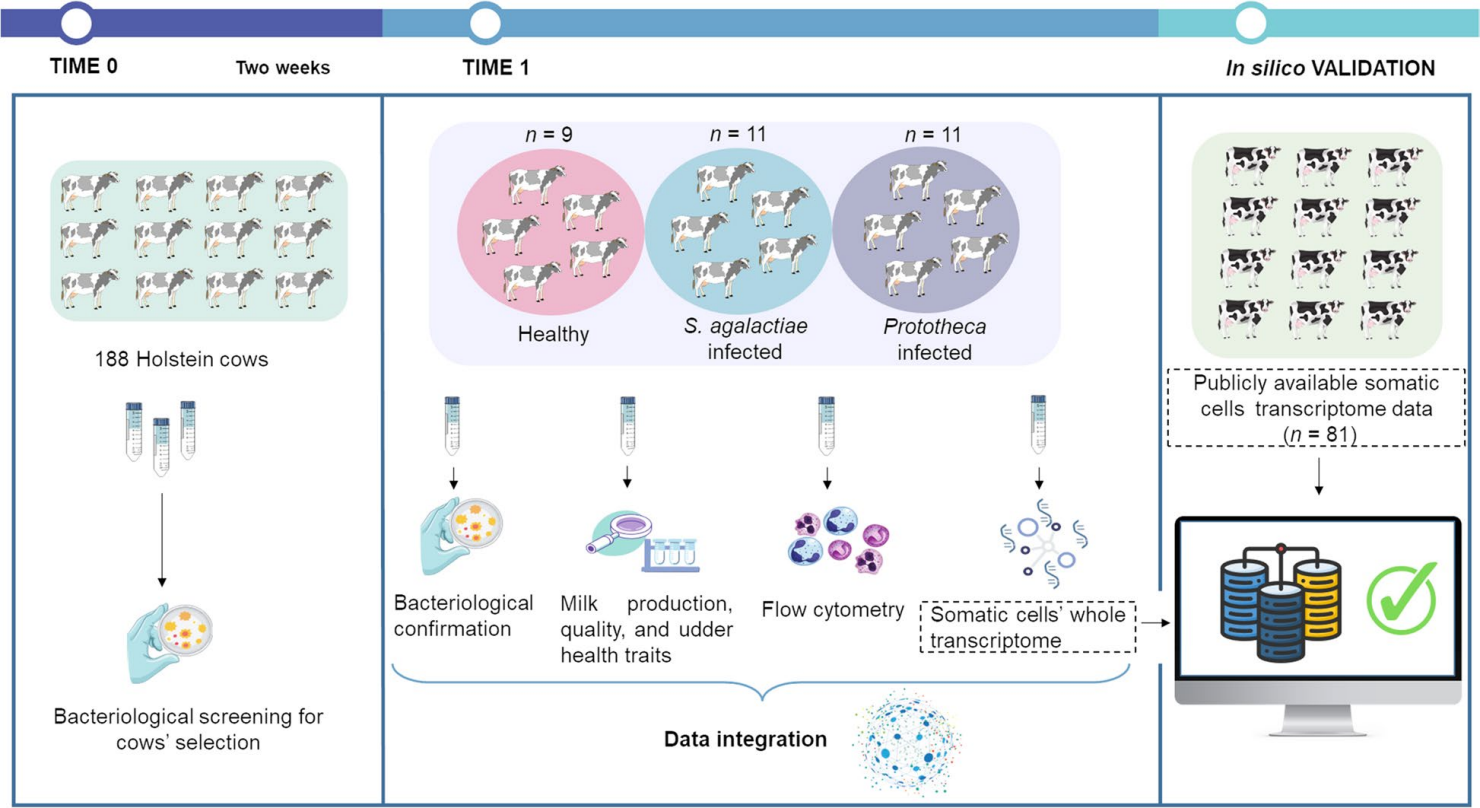
A schematic flow chart of the experimental design

The average milk yield was 26.97 (± 9.01) kg/d. Milk had 2.19 ± 0.74% of fat, 3.49 ± 0.29% of protein, 2.73 ± 0.27% of casein, and 4.51 ± 0.44% of lactose. Milk pH and conductivity were 6.46 ± 0.08 and 9.94 ± 1.30 mS/cm, respectively (Table S1). A schematic summary of the experimental design and the conducted analyses is reported in Fig. 1.

### *S. agalactiae* and *Prototheca* infections induced milk quality changes and divergent infiltration of immune cells

*S. agalactiae* and *Prototheca* infections were not accompanied by any clinical sign (e.g., udder swelling, redness, udder pain) or systemic reaction, but a significant drop in milk production (*P* < 0.05*)* compared to their uninfected counterparts (Fig. S1a). However, in contrast to the bacterial pathogen, *Prototheca* infection affected the milk quality more by reducing the lactose content (*P* < 0.05; Fig. S1b) and casein index (*P* < 0.05; Fig. S1c). Additionally, algal infection increased milk conductivity (*P* < 0.05; Fig. S1d), mirroring possible changes in the blood-milk barrier permeability. The milk protein, casein, and fat proportions were similar between groups (Fig. S1e–g) and urea concentration and milk pH (Fig. S1h and i). Both types of pathogens, however, increased the amounts of somatic cells (SC, *P* < 0.001; Fig. S2a) and the combined ratio of neutrophils and lymphocytes to total SCC (DSCC) compared to healthy animals (*P* < 0.05; Fig. S2b), reflecting the inflammatory status of the mammary gland. Although both pathogens increased the total leucocyte population (*P* < 0.001, Fig. S2c), the immunological cell content differed between *S. agalactiae* and *Prototheca* pathogens, evoking distinctly different immune responses to both pathogens. For instance, exposure to *S. agalactiae* primarily triggered the recruitment of nearby macrophages that increased more than 1.55-fold than the healthy animals. In contrast, *Prototheca* decreased macrophage populations by 20% (Fig. S2d) and sharply increased T helper cells (+ 73%), T killer cells (+ 110%), and B cells (+ 30%) compared to *S. agalactiae* (Fig. S2 e–g). No significant differences were found among the three experimental groups for PMN cells (Fig. S2h), even if their proportion was slightly higher in the *S. agalactiae*-induced mastitis. Importantly, we found large variability of immune cell contents among individuals within each group, as assessed by the principal component analysis (PCA) visualization (Fig. S2i).

### Somatic cell transcriptome changes upon *Prototheca *spp. and *S. agalactiae* infections

A total of seven billion paired-end reads were obtained from the somatic cells of 31 dairy cows (9 healthy and 22 naturally infected subjects), corresponding to an average of 119 M ± 49.8 M per sample. After quality filtering, 88.84% of high-quality paired reads were mapped, on average, to the bovine reference genome ARS-UCD1.2 and aligned with 27,607 unique genes. After filtering for genes with CPM > 0.5 in at least two samples, we obtained 14,564 abundant genes, henceforth referred to as expressed genes, corresponding to ~ 53% of the transcriptome (Table S3).

**Fig. 2:**
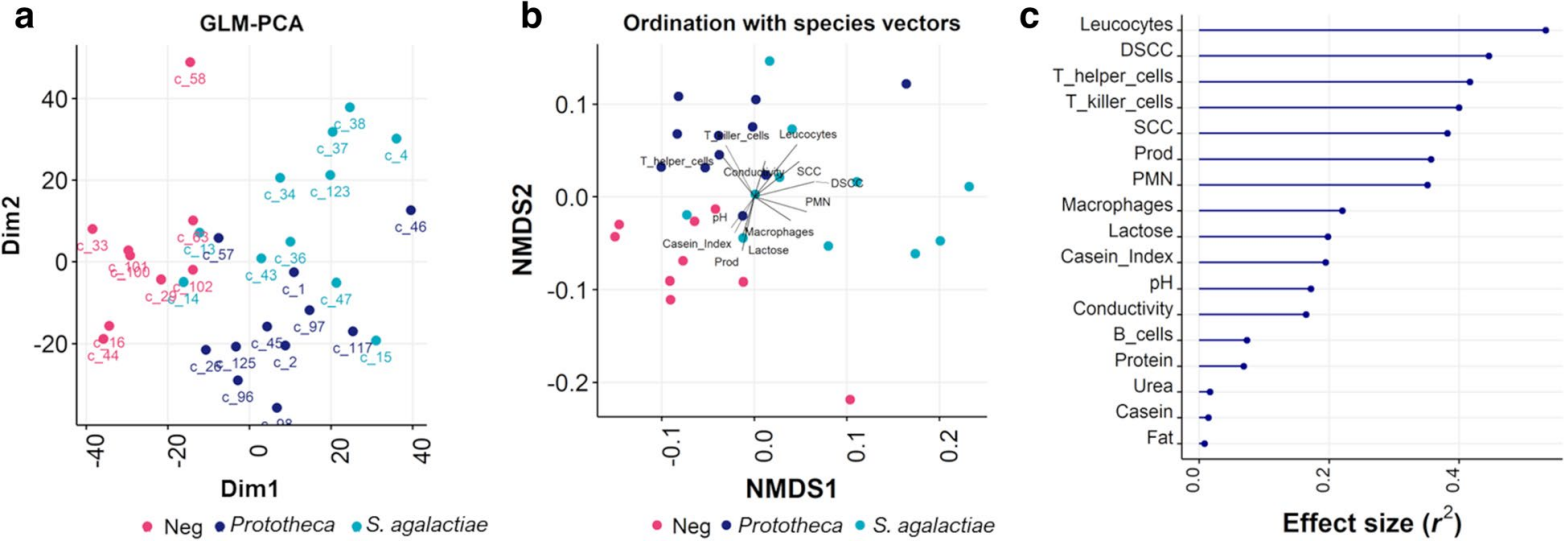
Plots of samples spatial separation. **a** Principal component analysis (PCA) of the 14,564 expressed genes; **b** Non-metric multidimensional scaling (NMDS) for the visualization of the variation of the 14,564 expressed genes according to the phenotypic traits; **c** The principal contributors explaining the total variance of the transcriptome. Dim1: dimension 1; Dim2: dimension 2; DSCC: differential somatic cell count; SCC: somatic cell count; prod: milk yield (kg/d)

The generalized PCA showed that the expression of genes varied according to the infection status, separating infected and uninfected individuals (generalized PCA axis 1: 22%; Fig. 2a). Altogether, transcriptome signatures fell into different *S. agalactiae, Prototheca,* and control response patterns, except for two *S. agalactiae* and one *Prototheca* spp. samples that grouped with healthy ones. Besides pathogen infection, we determined to what extent differences in host-associated variables could further explain the observed patterns of transcriptional variation. Using NMDS ordinations (Fig. 2b) for visualizing the structure of gene expression (ordination stress = 12.36%, *k* = 2, non-metric fit *r*^2^ = 0.985, linear fit *r*^2^ = 0.932), the principal contributors explaining the total variance of the transcriptome were flow cytometry variables, including leucocytes (*envfit*, *r*^2^ = 0.5329, *P* < 0.001), T-helper (*envfit*, *r*^2^ = 0.4167, *P* < 0.001), T-killer (*envfit*, *r*^2^ = 0.3999, *P* < 0.001) cells, and PMN (*envfit*, *r*^2^ = 0.3512, *P* < 0.01), together with udder health parameters, such as DSCC (*envfit*, *r*^2^ = 0.4456, *P* < 0.001) and SCC (*envfit*, *r*^2^ = 0.4456, *P* < 0.001), and milk yield (*envfit*, *r*^2^ = 0.3569, *P* < 0.01) (Fig. 2c). Macrophages, lactose, casein index, pH, and conductivity also accounted for the transcriptome variation, albeit of lesser significance.

A total of 1,682 DEGs were detected in *Prototheca-*infected animals (671 downregulated, 1,011 upregulated; *P* < 0.05) compared to the healthy controls, and these differences were even more significant for the *S. agalactiae-*infected group (*n* = 2,427 DEGs; 890 down- and 1,537 up-regulated; *P* < 0.05). DEG lists shared significant similarities between the two types of pathogens, as 40% of *Prototheca*’s DEGs were also expressed in the *S. agalactiae* group. When comparing *S. agalactiae* with *Prototheca* infections, 974 genes were differentially expressed (378 under and 596 over-expressed genes; *P* < 0.05). The list of all the DEGs belonging to each comparison is reported in Table S4. To gain insights into the biological and immune processes in response to the type of infection, we performed a functional enrichment analysis of the differentially up and downregulated genes.

**Fig. 3:**
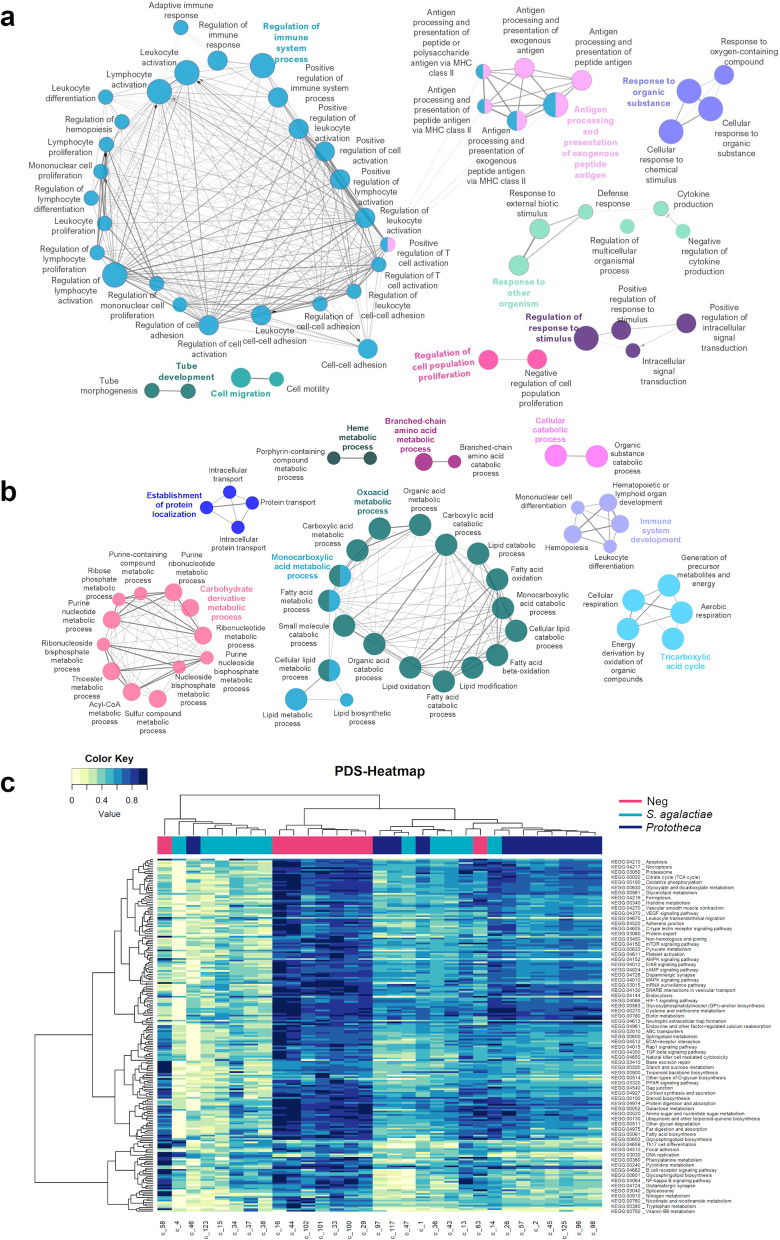
Pathway analysis of the differentially expressed genes in milk somatic cells. **a** ClueGO functionally grouped network of up and downregulated genes within the healthy and *Prototheca*’s infected cows. **b** ClueGO functionally grouped network of up and downregulated genes within the healthy and *S. agalactiae*’s infected cows. Terms are represented as nodes linked according to a kappa score ≥ 0.4. The node size means the term enrichment significance. Functionally related groups partially overlap. **c** Heatmap built on the pathway deregulation scores (PDSs) of the whole transcriptomic data of healthy, *Prototheca*’s, and *S. agalactiae*’s infected animals. Each row corresponds to a pathway, and each column to a sample. Blue corresponds to “no deregulation”, and yellow to high deregulation

Following *Prototheca* infection, 33% of DEGs were involved in immune system response (*n* = 228), antigen processing (*n* = 41), and response to other organisms’ invasion (*n* = 293) (Fig. 3a). Immune activation involved pathways related to innate response, such as toll-like receptors (*TLR9*) and pro-inflammatory molecules like IL-15, IL-17A, and IL-17F and adaptive immune response. The adaptive immune response mainly guided the defense line against *Prototheca* through the activation of class II MHC molecules (*BoLA-DMA*, *BoLA-DMB*, *BoLA-DOA*, *BoLA-DOB*, *BoLA-DRA*, *BoLA-DRB3),* stimulation of IFN-γ and IL4I1 (interleukin 4 induced 1), and proliferation of lymphocytes (*n* = 86 genes). The induction of *CD48* and *CD80* promoted T cell activation following *Prototheca* infection. At the same time, B lymphocyte upregulation was conducted by B lymphocyte differentiation (*IKZF3*) and B-cells response to antigens (*POU2AF1*).

In the context of *S. agalactiae* infection, the innate immune cells development and differentiation (*n* = 137) appeared as the first line of defense against the disease, coupled with a reduction of mitochondrial energy metabolism (*n* = 526, 21% of DEGs), that is, depletion of the TCA (*n* = 83), oxoacid (*n* = 266), and carbohydrate derivative metabolic processes (*n* = 177) (Fig. 3b). At a closer look, the immune response to *S. agalactiae* infection seemed to be led by Notch receptor 1 (*NOTCH1*), NF-κB signaling pathway, and pattern recognition receptors (PRR) like *TLR9* and *NOD2.* Other pathways involved in cell–cell adhesion (*SELL*, *CD274, BOLA-DRA, CSF3R*), pro-inflammatory chemokines, and cytokine secretion (*IL-17A*, *IL-17F*, *OSM*, *LTA*, *LTB*) and activation of the complement system C3 mediated were also enriched. Additionally, *S. agalactiae* modulated the expression of myeloid-derived suppressor cells (MDSCs) by activating the transcription factor *STAT3.* Despite the high number of differentially expressed genes in comparing the two pathogen infections (*S. agalactiae* vs. *Prototheca*; Table S4), no differential enriched pathways resulted from the analysis.

Last, we sought to support further that *S. agalactiae* and *Prototheca* triggered different immune responses using Pathifier. Through this algorithm, we identified 69 KEGG pathways with Pathway Deregulation Scores (PDS) significantly associated with the two types of infection compared to healthy individuals (Fig. 3c). Notably, the peroxisome proliferator-activated receptor (PPAR) pathway, an essential modulator of the immune response, was firmly (more than twofold) deregulated in *S. agalactiae*-infected animals compared to healthy counterparts. Additionally, NK cells mediated cytotoxicity path, which can be considered a more innate defense, was almost two times more deregulated in the *S. agalactiae* infection concerning the healthy animals. The energy-related paths (e.g., TCA cycle, carbohydrate metabolism, oxidative phosphorylation) in the *S. agalactiae* infection resulted in twofold deregulation compared to the healthy animals. At the same time, no significant alterations were detected for *Prototheca*’s infected animals.

Specific alteration in *Prothotheca*’s PDS involved a clear focal adhesion path, almost threefold lower than healthy animals and more than twofold lower than *S. agalactiae*. Conversely, the adherens junction path was specifically deregulated in *S. agalactiae* infection. Both disorders' pathways involving extracellular matrix receptor interaction (ECM) and gap junction were mildly deregulated. A closer look at the immune paths showed that both infections displayed strong PDS concerning the NF-κB signaling pathway. Despite it, the *S. agalactiae* infection showed the most diverging scores, especially when considering innate response-related functions such as leucocyte migration, endocytosis, and neutrophil extracellular trap formation. Interestingly, PDS concerning Th17 cell differentiation and B cell receptor signaling, pathways more explicit for the adaptive immune response, were significantly deregulated (more than twofold) in *Prototheca-*infected animals, even if a modest alteration was also observed in *S. aglactiae* infection.

### A set of core mastitis-response genes

Even if there were differences in intensity between the pathways regulated by the two microorganisms, the core immune transcriptome did not seem to respond so differently. For this reason, beyond the type of pathogen, we identified 1,954 DEGs in response to mastitis, of which 1,289 were under-expressed and 665 were over-expressed in infected individuals compared to healthy ones. Among them, 681 DEGs were commonly shared with the pathogen-specific DEGs lists. These were considered the core mastitis-response genes (Table S4). Enzymes make up the most significant gene function category (67%), outranking transcription factors (TF, 8.8%), transporters (6.7%), transmembrane receptors (4.4%), kinases (3.8%), and G-protein coupled receptors (2.5%). Around 9.8% of genes were unannotated. Two hundred twenty-nine genes were highly expressed in infected samples. The functional analysis showed that roughly 23% of these genes (*n* = 156) were directly involved in activating and regulating the immune response. In contrast, 69 genes were associated explicitly with catabolic and oxidative pathways (Fig. S3). Interestingly, inflammasome activation and regulation were enriched upon encountering a pathogenic agent (e.g., *NLRC5, TLR9, GBP5, PLCG2*) and the mitochondrial-related genes (*n* = 80; hypergeometric test, an adjusted *P*-value of 5.29 × 10^–5^). Moreover, we found the downregulation of pathways involved in the lipid metabolism and synthesis of de-novo fatty acids (*FASN*, *ACACA*).

### Integration of core mastitis-response genes and phenomic data

**Fig. 4:**
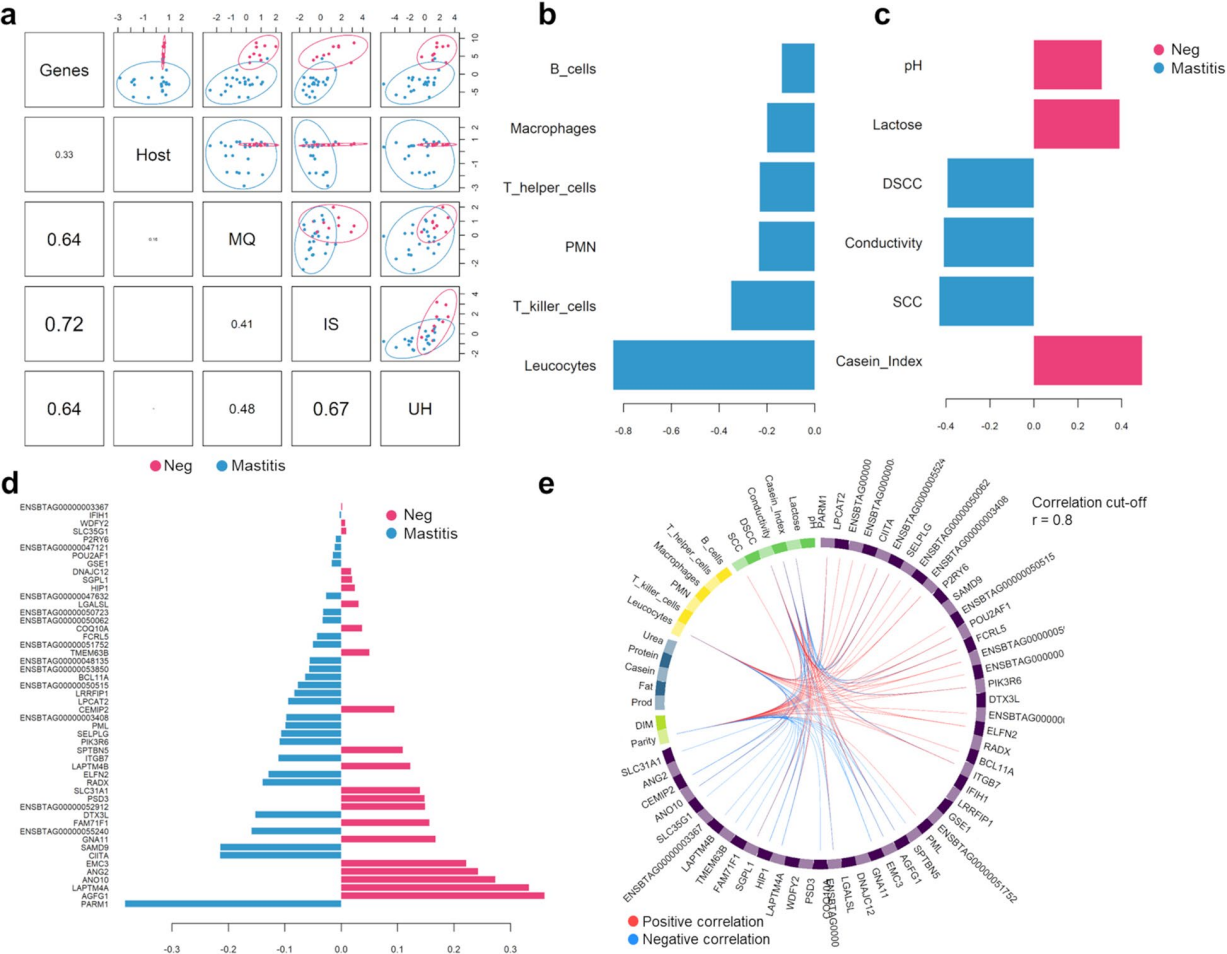
Integration of transcriptomic and phenomic information using the DIABLO approach. **a** Correlation plot among the different sets of categories; **b–d** The loading plots of flow cytometry immune cells (**b**) udder health traits (**c**) and differentially expressed genes (DEGs) (**d**). Pink and light blue bars represent healthy animals and cows with subclinical intramammary infection (sIMI), respectively. The negative values of the loading weights (light blue bars) signify that the corresponding variables had higher expression/value in infected animals. The positive values (pink bars) mean that related variables had higher expression/values in healthy animals. **e** Circos plot showing the correlation between candidate variables. MQ: milk quality, IS: immune system, UH: udder health

Using DIABLO, we observed the strongest covariation between the core mastitis genes and the immune cells populations (IS) (*r*^2^ = 0.72), followed by the udder health (UH) (*r*^2^ = 0.64) and milk quality (MQ) parameters (*r*^2^ = 0.64; Fig. 4a). No important covariation was found between the core mastitis response genes and the host variables (e.g., parity and DIM; *r*^2^ = 0.33). Concomitantly, the immune cells co-varied with the udder health-related variables (*r*^2^ = 0.64). Then, to add biological meaning to the predicted model, we investigated the relationship between the DIABLO-selected features with the highest covariation. The first latent variable of the immune cells data set supported induction of the immune system response in mastitis cows, with increased infiltrations of leucocytes and T-killer cells, and to less extent, PMN, T-helper cells, macrophages, and B cells (Fig. 4b). Paired with these immune-related cells, the first latent variable for the udder health parameters pointed at higher levels of DSCC, SCC, and milk conductivity upon mastitis but lower casein index, lactose, and pH (Fig. 4c). Regarding the genes selected, the first latent variable of the expected model indicated that subjects with mastitis overexpressed the prostate androgen-regulated mucin-like protein 1 (*PARM1)* gene. Moreover, in infected animals, we observed the induction of genes involved in the transcription of class II MHC molecules (*CIITA*), cell proliferation and apoptosis (*SAMD9*), and adhesion and diapedesis of granulocytes (*SELPLG*) (Fig. 4d). Conversely, healthy subjects were primarily defined by genes related to molecules transportation and transmembrane proteins (*LAPTM4A*, *ANO10*, *GNA11*).

### Identification of mastitis hub variables

The co-association of gene expression and phenomic data obtained from CIRCOS (Fig. 4e) resulted in a network construction consisting of 116 interactors (nodes) and 4,430 interactions (edges). This network was further analyzed using the Molecular Complex Detection (MCODE) Cytoscape plug-in, which identified four densely connected modules. The two most significant modules showed MCODE scores of 54.732 and 6.667, respectively. Seven variables (*AGFG1*, *CEMIP2*, *ITGB7*, *RRAD*, Urea, T killer cells, and leucocytes) were not assigned to any module (Fig. S4).

**Fig. 5:**
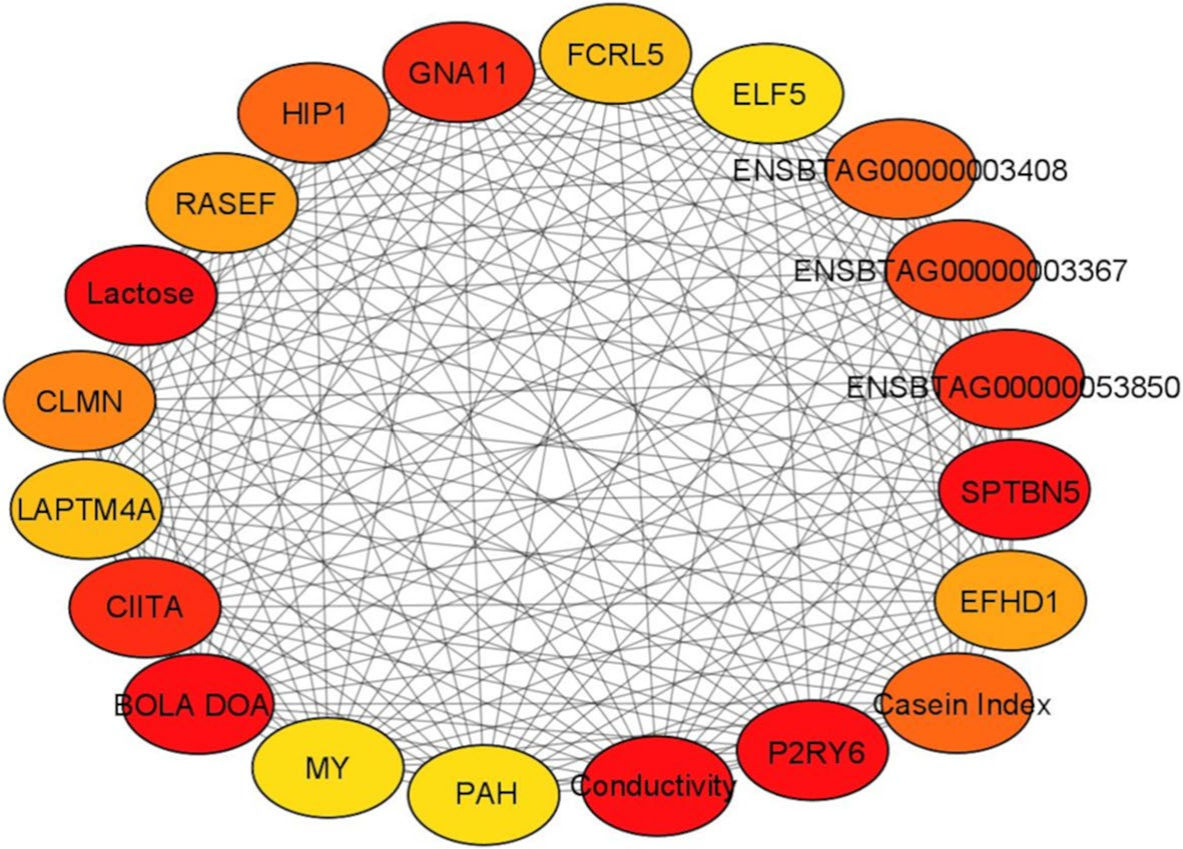
Cytohubba top 20 hub variables according to the maximal clique centrality method (MCC). Higher ranking is represented by a redder color

Lastly, with cytoHubba, we identified the top 20 hub variables, which are displayed in Fig. 5 and ordered as follows according to the MCC ranking method: milk conductivity, lactose, *P2RY6*, *SPTBN5*, *BoLA-DOA*, *ENSBTAG00000053850*, *CIITA*, *GNA11*, *ENSBTAG0000003367*, casein index, *ENSBTAG0000003408*, *HIP1*, *CLMN*, *RESEF*, *EFHD1*, *LAPTM4A1*, *FCRL5*, milk yield, *PAH*, *ELF5* (Full MCC ranking in Table S5). The genes that were commonly shared by both DIABLO and cytoHubba (*P2RY6, SPTBN5, BoLA-DOA, CIITA, GNA11, ENSBTAG0000003367, ENSBTAG0000003408, HIP1, LAPTM4A, FCRL5*) were then submitted to a ROC analysis resulted in having excellent prediction performances in terms of discriminating healthy and mastitis animals with sensitivity > 0.89, specificity > 0.81, accuracy > 0.87 and precision > 0.69. The detailed predictive performances of the hub genes are presented in Table S6.

### Meta-analysis of subclinical mastitis

**Fig. 6:**
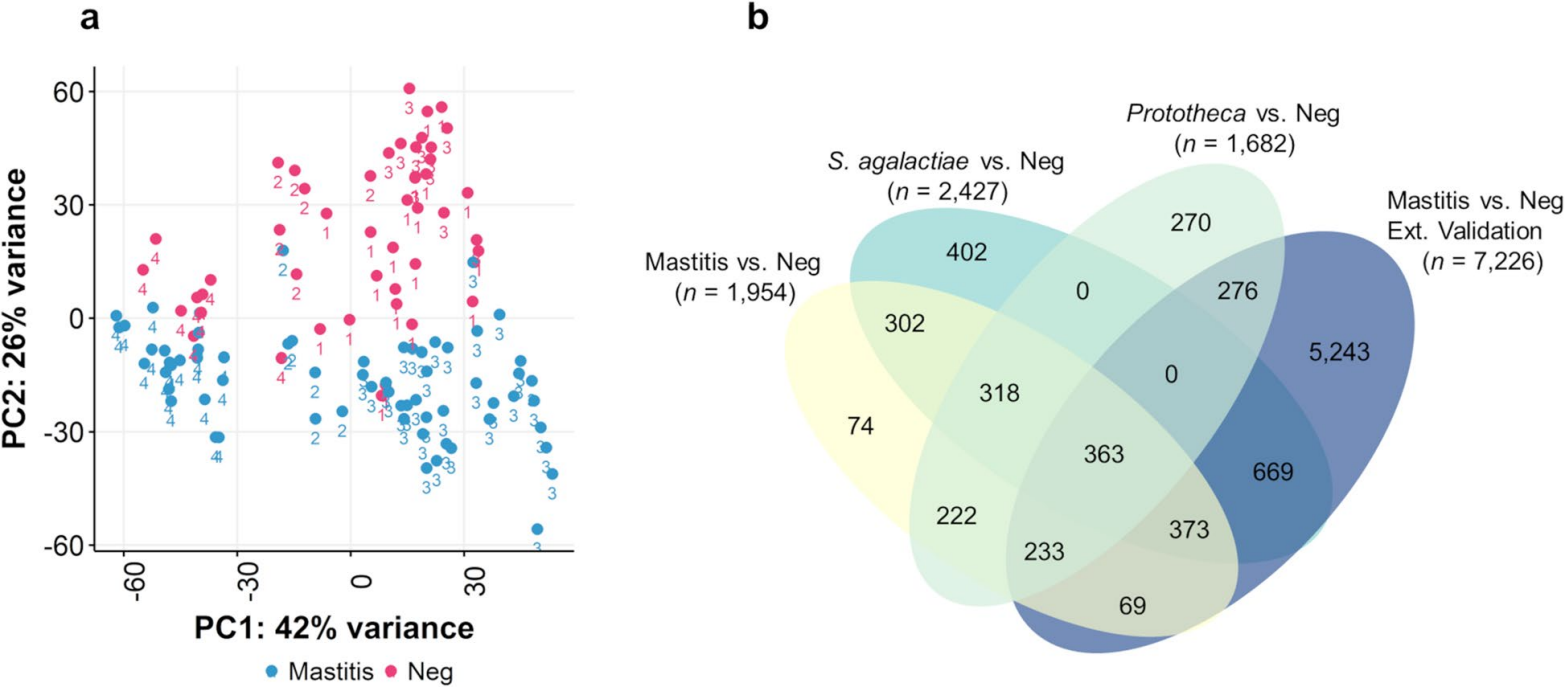
The meta-analysis. **a** Principal component analysis (PCA) of the present dataset and the ones downloaded from public repositories on milk somatic cell transcriptome. **b** Venn diagram of differentially expressed genes (DEGs) among the different experimental comparisons (*Prototheca* vs*.* healthy, *S. agalactiae* vs*.* healthy, mastitis vs*.* healthy, mastitis vs*.* healthy external data set). 1: transcriptomic data from the work of Seo et al. [38]; 2: transcriptomic data from the work of Asselstine et al. [14]; 3: transcriptomic data from the work of Niedziela et al. [15]; 4: transcriptomic data from the present dataset

To validate the core mastitis response genes, we additionally gather publicly available somatic cell transcriptome RNA-seq datasets (*n* = 81) derived from three independent studies in dairy cows: i) pooled milk sampled from 12 healthy cows [38], ii) quarter samples from 6 cows (*n* = 12) [14], and iii) 14 cows sampled at five different times (*n* = 48) [15]. As with the study cohort, the transcriptome profiles could be distinguished based on the infection status (Fig. 6a), with higher transcriptome dispersion in infected individuals. The first principal component accounted for 51% of the total variance, and the first two accounted for 74%. Consistent with the training set, many genes (*n* = 7,226) were differentially expressed between the healthy and infected individuals, including 3,912 up- and 3,314 down-regulated. We then intersected the list of core mastitis-response genes and the list of DEGs from these published datasets (Fig. 6b). We found that 53% of these core-mastitis response genes were DEG in the validation set, strengthening the shared transcriptional response to infection, independently on the pathogen, regional, and other potential differences, such as diet, medication, energy expenditure, age, and DIM of the dairy cows.

To further assess the feasibility of the previously selected hub genes for discriminating healthy and infected animals, we re-performed a ROC analysis on the unified training-validation dataset (*n* = 112). The class II transactivator (*CIITA*) had the best prediction performances, having sensitivity, specificity, and accuracy > 0.7 and precision equal to 0.8.

## Discussion

Somatic cells are released in milk as the first line of defense against mammary infections, and they are widely applied as an indicator for mastitis screening and detection. Moreover, their expression signatures fit suitably for performing mastitis mammary-gland-specific studies and monitoring the pathogen-specific molecular response.

To the best of our knowledge, this is the first study on the milk somatic cell transcriptome variations in response to the infection of two pathogens: *S. agalactiae*, a Gram-positive bacterium, and *Prototheca* spp., a microscopic alga. *Prototheca*’s molecular mechanism of action is still poorly understood and has only recently been recognized as a non-negligible mastitis agent, with an estimated 11.2% prevalence on Italian territory [6]. In addition, for the first time, we integrated somatic cell transcriptome data with a wide range of phenotypic traits in a joint analysis to identify hub variables affected by subclinical intramammary infection in dairy cattle.

We tried to minimize any possible source of variation that might modify the transcriptional response by excluding primiparous cows and animals during their periparturient period. In fact, in early lactating cows, the decreased feed intake and the increased energy demand often result in an energy-negative balance condition [39], which can also affect the proper activation of the immune cell’s metabolism in response to pathogens' invasion [40].

In terms of production performances, the significant drop in milk yield in the infected animals (regardless of the pathogen) was in line with what was observed by previous studies conducted on subclinical mastitis [41]. Instead, the most quality-impaired milk was the one from *Prototheca* infection. Pegolo et al. [42] reported similar results on a broader cohort of animals affected by subclinical IMI from four pathogens: *S. aureus*, *S. agalactiae*, *S. uberis,* and *Prototheca* spp. Both pathogens significantly increased milk SCC compared to the negative control samples but resulted in different leucocyte proportions. Exposure to *S. agalactiae* primarily triggered an innate immune response by recruiting nearby macrophages at the site of infection. At the same time, *Prototheca* seemed to show resistance to the phagosomal defense mechanism and a more adaptive-driven response through the crucial role of T-cells. Even though the diverse leucocyte profile suggests a different immune response, the large variability of the immune cells assessed by the PCA (Fig. S2i) evokes that beyond infection and protection, immune cell patterns could also be driven by a range of genetic and non-genetic factors such as the inflammation stage as well as individual environmental exposures.

### SC transcriptome response upon *S. agalactiae* and *Prototheca* infection

To further dissect these phenotypic phenomena, the RNAseq approach was applied. Antigen-presenting and processing was a critical pathway enriched in *Prototheca* spp. infected samples, suggesting that the adaptive immune response was mainly guided by the activation of MHC class II molecules. The bovine MHC genes are called the bovine lymphocyte antigen (*BoLA*) [43]. Specifically, class II is expressed on antigen-presenting cells and activates CD4^+^ T cells, resulting in the coordination and regulation of effector cells [44].

*S. agalactiae* was found to modulate the expression of MDSCs, a heterogeneous subset of immature monocytes and granulocytes, by activating *STAT3*, which stimulates myelopoiesis, inhibiting myeloid-cell differentiation [45]. Interestingly, the infection of *S. agalactiae* induced a down-regulation of mitochondrial energy related-pathways like the TCA cycle, oxoacid, and carbohydrate derivative metabolic processes, which might be partially linked to the relationship existing between macrophages and mitochondria. With the onset of inflammation, macrophages activate, showing a proinflammatory profile metabolically characterized by an increased glycolysis and lactate production [46]. In contrast, even in the presence of oxygen, mitochondrial oxidative phosphorylation (OXPHOS) is reduced in pro-inflammatory macrophages (the so-called Warburg effect), presumably as an effect of the tricarboxylic acid (TCA) cycle fragmentation [47].

Finally, using the Pathifier algorithm, we identified 69 KEGG pathways which explained the differential gene expression profile in the two types of infection for the negative control. In line with our previous findings, we observed the significant deregulation in the TCA cycle, carbohydrate metabolism, oxidative phosphorylation pathways as well as the peroxisome proliferator-activated receptor (PPAR), which is an essential modulator of the immune response tightly linked to mitochondrial metabolism [48], confirmed the role of mitochondria as major hubs in inflammatory and immune response carried out by *S. agalactiae*.

Pathways involving ECM, focal adhesion, and gap junction, which comprehend the group of genes involved in the communication and integrity of the epithelial cells [49], were mildly deregulated in both infections, confirming transcriptomic and iTRAQ-proteomics patterns on Chinese Holstein cows challenged with *S. agalactiae* via nipple tube perfusion [50]. Altered gene expression within these pathways might be related to the reorganization of the actin cytoskeleton, which may represent one way of reducing tissue damage caused by invading pathogens. Our previous functional analysis supported these findings in which we found some integrin family coding genes. In *S. agalactiae*-positive samples, we found *ITGA5* upregulated, similar to the work of Niedziela et al. [15]. This gene encodes the light and heavy chains that create the α5 subunit, which, by associating with the β1 subunit, form the receptors for fibronectin and fibrinogen. These two glycoproteins are essential mediators of the pathogen adhesion [51]. Their abundance increased in the work of Mudaliar et al. [52], where a label-free proteomic approach analyzed the changes in the protein profile of milk whey in a cohort of animals experimentally infected by *S. uberis.* In *Prototheca* infection, we found instead *ITGA9*, which encodes integrin subunit β7, necessary for the leukocyte adhesion [53].

Moreover, PDS concerning Th17 cell differentiation and B cell receptor signaling, pathways more specific for the adaptive immune response, were significantly deregulated in *Prototheca-*infected animals, even if a modest alteration was observed in *S. agalactiae* infection.

It is crucial to notice that with this approach, we also found significant differences in PDS within samples belonging to the same group. This is partially linked to the fact that we did not have the species identification for *Prototheca* or the strain identification for *S. agalactiae*, which led to divergent responses. Secondly, as we worked on naturally occurring mastitis, we could have had different stages of inflammation in our samples.

### Common transcriptomic signature of subclinical intramammary infection

Despite some differences in the DEGs expression within the activation of the immune system pathways, we did not find extreme differences in the two microorganisms’ transcriptomic profiles, despite the great phylogenetic distance between them. This was further confirmed by the enrichment analysis comparing the DEGs among the two pathogens that did not produce significant pathways. It is, however, important to remember that *S. agalactiae* and *Prototheca* are known to induce a weak immune reaction compared to Gram-negative bacteria like *E. coli*, which results in subclinical mastitis with usually no systemic repercussions [10]. Both pathogens can put in place mechanisms of immune evasion that might explain a more moderate inflammatory response. Indeed, in *S. agalactiae*, synthetase proteins such as FbsA and FbsB are involved in fibrinogen binding that can decrease the risk of opsonization by phagocytic cells. Moreover, the production of the serine protease CspA allows this pathogen to further evade the immune response by cleaving specific chemokines responsible for the neutrophil recruitment [54]. *Prototheca*, conversely, seems to be able to form a biofilm which could be implicated in its pathogenicity, partial immune invasion [11], and ability to persist in the environment [55].

Regardless of the pathogen, we identified several highly expressed genes in animal samples with subclinical IMI. This suggests these “core mastitis-response genes” may represent a typical infection signature and provide a potential therapeutic window for mastitis drug development.

Pathway analysis conducted on the 681 core-mastitis response genes (the ones commonly shared by the two infections) identified that most upregulated pathways were related to the activation of innate and adaptive immune system processes. Interestingly, among the expressed ILs, the significant upregulation of *IL-17A* and *IL-17F* might be specifically linked to the so-called “type 3” immunity, which encompasses innate and adaptive immune response and is characterized both by the recruitment of neutrophils and the stimulation of epithelial antimicrobial defenses at the sites of infection [56]. Overexpression of *IL-17A* and *IL-17F* encoding genes was also observed in the mammary gland of cows infected with *E. coli* [57] and in goats infected by *S. aureus* [58]. In contrast, the downregulated ones involved oxoacid metabolic processes. These biological processes were commonly shared with the work of Asselstine et al. [14], one of the few published papers that characterized the milk somatic cell transcriptome in Holstein cows. In addition, they found several unspecific Gene Ontology (GO) terms, which might be explained by the fact that genes expressing alternative transcripts might have been associated with a heterogeneous role in biological functions [59].

*NLRC5*, *TLR9*, *GBP5*, and *PLG2* were highly enriched upon encountering the pathogenic agents. These genes are well-known to have a pivotal role in the activation of *NLRP3* (NLR family PYD containing 3), which is considered one of the essential activators and synergic components of the inflammasome [60] which, in turn, are a class of molecules assembled by the PRRs which are well known to play an important role in innate immunity through the stimulation of pro-inflammatory cytokines and pyroptosis [61]. Moreover, the identification of several mitochondrial-related DEGs was consistent with the hypothesis that mitochondria activity may be regulated by subclinical intramammary infection.

Within the pathway involved in the fatty acid metabolic process, we found several lipogenic genes (*FASN*, *ACACA*) that were downregulated. The decreased expression of these genes in animals with mastitis compared with negative control was also observed in the work of Moyes et al. [62] and Huma et al. [63] might probably be related to the fact that with the onset of inflammation, the energy demand needed to produce new fatty acids is too high [64].

Interestingly, the most under-expressed gene was the *SLC34A2*, according to what was observed by Asselstine et al. [14]. *SLC34A2* encodes the solute carrier family 34 members 2, a sodium-dependent phosphate transporter, which was upregulated in disease-resistant cows [65]. It is therefore considered a putative biomarker for selecting disease resistance in dairy cattle.

### Identification of hub variables for subclinical intramammary infection

Among the candidate DE genes identified by DIABLO, the class II transactivator (*CIITA*) had one of the highest loading scores and was significantly upregulated in mastitis animals. *CIITA* has been recognized as one of the master regulators in the gene expression of MHC. Moreover, it is involved in the transcription graduation of over 60 immunologically essential genes, including interleukin 4 (*IL-4*) and *IL-10* [66]. *CIITA* was found to be a critical regulator of the immune response of *Cynoglossus semilaevis* towards the infection of *Vibrio harvey*, suggesting its putative involvement also in the molecular inflammatory process of mastitis. Interestingly, it was also proposed as one of the most important candidate genes for bovine paratuberculosis tolerance in the GWAS study conducted by Canive et al. [67]

All the immune-cells variables selected by DIABLO were positively associated with mastitis, with total leucocytes having the highest loading score. It is already widely established for milk quality traits that SCC, and more recently DSCC, represent the most critical and easy-to-use indicator for identifying inflammation at the udder level [68, 69].

Moreover, lactose and casein index proportion are two traits that are highly influenced by inflammatory processes. The reduction in lactose proportion associated with clinical and subclinical mastitis is related to the reduced secretory activity of the mammary epithelial cells and an increase in the permeability of the mammary epithelium due to tight junction impairment [70]. The casein index reduction upon IMI is related to the increased proteolytic activity due to both endogenous and bacterial proteases that particularly damage the casein fractions [71, 72]. Casein index and lactose content represent a useful additional tool for discriminating against healthy/infected animals.

Among the phenotypic indicators identified by the integrated DIABLO-cytohubba approach, milk conductivity, and lactose were the ones showing the highest ranking. In fact, during the inflammation of the mammary gland, the osmotic balance is maintained through the increase in Na^+^ and Cl^−^, which are responsible for the rise in milk electrical conductivity [73]. In this context, a study by Ebrahimie et al. [74] on Holstein cows identified milk lactose and conductivity, together with SCC, as the most reliable indicators for the detection of subclinical mastitis. Moreover, also a recent work conducted by Antanaitis et al. [75] on 5,814 cows observed a strong association between lactose levels and subclinical mastitis pathogens, concluding that it might be helpful to include lactose (as well as milk conductivity) as a biomarker of suspected udder inflammation in health prevention programs.

Among the selected hub genes, *BoLA-DOA* encodes the major histocompatibility complex (MHC), class II, DO alpha. It is already well known that molecules linked to MHC play a fundamental role in the antigen recognition, presentations, and activation of the adaptive immune response [43]. Among the numerous molecules that belong to the BoLA family, *BoLA-DOA* specific mechanism of action has yet to be unraveled. Nonetheless, some studies found this gene upregulated in the presence of mastitis. Chen et al. [76], for example, in the transcriptional survey of exosomes derived from *Staphylococcus aureus*-infected bovine mammary epithelial cells, found a significant upregulation of the *BoLA-DOA* gene. Conversely, Cheng et al. [77] observed the downregulation of *BoLA-DOA* and several other genes involved in antigen presentation and processing in the blood-circulating leucocytes of animals recovering from *E. coli* clinical mastitis. This difference in gene expression might be related to the fact that *E. coli*, unlike *S. agalactia*e or *Prototheca*, generally induces an acute and robust udder inflammation with a more generalized immune response [8].

One of the essential hub genes downregulated in mastitis animals was *GNA11,* which encodes for a type of guanine nucleotide-binding protein (G-protein) functioning as a modulator or transducer in the transmembrane signaling systems. In the recent work of Pan et al. [78] on transcriptome evaluation in early calf nutrition, *GNA11* was significantly implicated in energy-related pathways, especially fat metabolism. Therefore, its downregulation in mastitis-positive samples should be further evaluated better to explain the relationships between energetic pathways and immune response.

## Conclusions

This work evaluated for the first time the somatic cell transcriptomic signature derived from naturally occurring subclinical mastitis caused by two different etiological agents: *S. agalactiae* and *Prototheca* spp. Even though we found some differences in the immune-related pathways and gene expression between the two infections (e.g., more robust activation of the antigen and processing complex in *Prototheca* and potent inhibition of energy-related pathways in *S. agalactiae* infection), the core immune response was commonly shared between the two pathogens.

The integrated analysis of core mastitis response genes and phenotypic traits confirmed a strong correlation between the transcriptome and the leucocyte populations determined by flow cytometry and with udder health traits (SCC, lactose, conductivity, and casein index), strengthening the need to systematically include them as screening and diagnostic indicator for the IMI detection. Finally, the predictive performances on the hub genes tested within the meta-analysis highlighted that *CIITA* might have a crucial role in the molecular mechanism underlying the animals’ response to subclinical IMI and need further evaluation in future studies, also taking into consideration a wider cohort of animals.

## Supplementary Information

**Additional file 1:** **Table S1.** Metadata of the study.

**Additional file 2:** **Table S2.** Read mapping statistics of the transcriptome data before and after trimming.Quality control has been conducted using multiQC.

**Additional file 3:** **Fig S1.** Variation of milk phenotypic traits in healthy, *Prototheca*’s and *S. agalactiae*’s infected animals. Boxplots of milk yield, milk lactose, milk casein index, milk conductivity, milk protein, milk casein, milk fat, milk urea and milk pHaccording to the three experimental groups.

**Additional file 4:** **Fig. S2.** Flow cytometry results for immune cell populations according to the three experimental groups. Violin plots of milk somatic cell score, differential somatic cell count, leucocytes, macrophages, T helper cells, T killer cells, B cellsand, PMNin healthy, *Prototheca’*s and *S. agalactiae’*s infected animals.Principal component analysis shows the samples’ separation according to the flow cytometry variables.

**Additional file 5:** **Table S3.** Matrix of the 14,564 expressed genes obtained after filtering for counts per million > 0.5 in at least 10 samples. Genes failing these criteria were removed from the exploratory and DEGs analyses.

**Additional file 6:** **Table S4.** List of the DEGs for each experimental comparison.

**Additional file 7:** **Fig. S3.** ClueGo pathway analysis of the 681 “core mastitis response genes” commonly shared between *S. agalactiae* and *Prototheca.*

**Additional file 8:** **Fig. S4.** PPI network construction and module analysis carried out with Cytoscape’s plug-in MCODE. Nodes belonging to different modules are differently colored. White nodes are variables that were not assigned to any modules. Lines represent the interaction between nodes.

**Additional file 9:** **Table S5.** Full Maximal Clique Centralityranking of the 20 hub variables obtained with cytohubba.

**Additional file 10:** **Table S6.** Predictive performances obtained through ROC analysis for the hub genes.

## Data Availability

The sequencing data of this study were deposited in the NCBI’s Sequence Read Archive (SRA) under the PRJNA911953 accession number. Public RNA-seq data were downloaded from the GEO database at GSE60575 accession number (Seo), from NCBI PRJNA544129 Bioproject accession number (Asselstine), and the European Nucleotide Archive (ENA) repository with the project number PRJEB43443 (Niedziela).

## Abbreviations

ACACA: Acetyl-CoA carboxylase alpha
ANO10: Anoctamin 10
BoLA-DOA: *Bos taurus* major histocompatibility complex, class II
*CIITA*: Class II major histocompatibility complex transactivator
CPM: Counts per million
DEG: Differentially expressed gene
DIABLO: Data Integration Analysis for Biomarker discovery using Latent Components
DIM: Days in milk
DSCC: Differential somatic cell count
*E. coli*: *Escherichia coli*
ECM: Extracellular matrix receptor interaction
ER: Endoplasmic reticulum
FASN: Fatty acid synthase
FDR: False discovery rate
GNA11: G protein subunit alpha 11
GO: Gene Ontology
GWAS: Genome-wide association study
HIP1: Huntingtin interacting protein 1
IFN- γ: Interferon gamma
IL-10: Interleukin10
IL-15: Interleukin 15
IL-17A: Interleukin 17A
IL-17F: Interleukin 17F
IL41I: Interleukin induced 1
IMI: Intra-mammary infection
IS: Immune cells
ITGA5: Integrin subunit alpha 5
ITGA9: Integrin subunit alpha 9
ITGB7: Integrin subunit beta 7
IZSVe: Istituto Zooprofilattico Sperimentale delle Venezie
KEGG: Kyoto Encyclopedia of Genes and Genomes
LAPTM4A: Lysosomal protein transmembrane 4 alpha
LTA: Lymphotoxin alpha
LTB: Lymphotoxin
MCC: Maximal Clique Centrality
MCODE: Molecular complex detection
MDSC: Myloid derived suppressor cells
MHC: Major histocompatibility complex
MQ: Milk quality
NF-κB: Nuclear factor κB
NK: Natural killer
NLRC5: NLR family CARD domain containing 5
NMDS: Non-parametric multidimensional scaling
NOD2: Nucleotide binding oligomerization domain containing 2
OSM: Oncostatin
OXPHOS: Mitochondrial oxidative phosphorylation
P2RY6: Pyrimidinergic Receptor P2Y6
PARM1: Prostate androgen-regulated mucine-like protein 1
PCA: Principal component analysis
PDS: Pathway deregulation scores
PPAR: Peroxisome proliferator-activated receptor
PRR: Pattern recognition receptor
RNA-Seq: RNA sequencing
ROC: Receiver operating characteristics
RRAD: Ras related glycolysis inhibitor and calcium channel regulator
*S. agalactiae*: *Streptococcus agalactiae*
*S. aureus*: *Staphylococcus aureus*
SAMD9: Sterile alpha motif domain containing 9
SC: Somatic cells
SCC: Somatic cell count
SELPLG: Selectin P ligand
SPTBN5: Spectrin beta, non-erythrocytic 5
STAT3: Signal transducer and activator of transcription 3
T0: Time 0
T1: Time 1
TCA: Tricarboxylic acid cycle
Th17: T helper 17 cells
TLR9: Toll-like receptor 9
UH: Udder health

## References

[1] Halasa T, Huijps K, Østerås O, Hogeveen H (2007). Economic effects of bovine mastitis and mastitis management: a review. Vet Q.

[2] Bradley AJ (2002). Bovine mastitis: an evolving disease. Vet J.

[3] Martin P, Barkema HW, Brito LF, Narayana SG, Miglior F (2018). Symposium review: novel strategies to genetically improve mastitis resistance in dairy cattle. J Dairy Sci.

[4] Ruegg PL (2017). A 100-Year Review: Mastitis detection, management, and prevention. J Dairy Sci.

[5] Ranjan R, Swarup D, Patra RC, Nandi D. Bovine protothecal mastitis: a review. CAB Rev Perspect Agric Vet Sci Nutr Nat Resour. 2006;1:1–7. 10.1079/PAVSNNR20061017.

[6] Shave CD, Millyard L, May RC (2021). Now for something completely different: *Prototheca*, pathogenic algae. PLoS Pathog.

[7] Schukken YH, Günther J, Fitzpatrick J, Fontaine MC, Goetze L, Holst O (2011). Host-response patterns of intramammary infections in dairy cows. Vet Immunol Immunopathol.

[8] Petzl W, Zerbe H, Günther J, Seyfert HM, Hussen J, Schuberth HJ (2018). Pathogen-specific responses in the bovine udder. Models and immunoprophylactic concepts. Res Vet Sci.

[9] Jensen K, Günther J, Talbot R, Petzl W, Zerbe H, Schuberth HJ, et al. *Escherichia coli*- and *Staphylococcus aureus*-induced mastitis differentially modulate transcriptional responses in neighbouring uninfected bovine mammary gland quarters. BMC Genomics. 2013;14:36. 10.1186/1471-2164-14-36. 10.1186/1471-2164-14-36 PMC3598231 23324411

[10] Günther J, Petzl W, Bauer I, Ponsuksili S, Zerbe H, Schuberth HJ, et al. Differentiating *Staphylococcus aureus* from *Escherichia coli* mastitis: *S. aureus* triggers unbalanced immune-dampening and host cell invasion immediately after udder infection. Sci Rep. 2017;7:4811.10.1038/s41598-017-05107-4. 10.1038/s41598-017-05107-4 PMC5500526 28684793

[11] Pegolo S, Toscano A, Bisutti V, Giannuzzi D, Vanzin A, Lisuzzo A, et al. *Streptococcus agalactiae* and Prototheca spp. induce different mammary gland leukocyte responses in Holstein cows. JDS Commun. 2022;3:270–4. 10.3168/jdsc.2022-0216. 10.3168/jdsc.2022-0216 PMC9623724 36338024

[12] Wang D, Liu L, Augustino SMA, Duan T, Hall TJ, MacHugh DE, et al. Identification of novel molecular markers of mastitis caused by *Staphylococcus aureus* using gene expression profiling in two consecutive generations of Chinese Holstein dairy cattle. J Anim Sci Biotechnol. 2020;11:98. 10.1186/s40104-020-00494-7. 10.1186/s40104-020-00494-7 PMC7488426 32944235

[13] Heimes A, Brodhagen J, Weikard R, Seyfert HM, Becker D, Meyerholz MM, et al. Hepatic transcriptome analysis identifies divergent pathogen-specific targeting-strategies to modulate the innate immune system in response to intramammary infection. Front Immunol. 2020;11:715. 10.3389/fimmu.2020.00715. 10.3389/fimmu.2020.00715 PMC7202451 32411137

[14] Asselstine V, Miglior F, Suárez-Vega A, Fonseca PAS, Mallard B, Karrow N (2019). Genetic mechanisms regulating the host response during mastitis. J Dairy Sci.

[15] Niedziela DA, Cormican P, Foucras G, Leonard FC, Keane OM. Bovine milk somatic cell transcriptomic response to *Staphylococcus aureus* is dependent on strain genotype. BMC Genomics. 2021;22:796. 10.1186/s12864-021-08135-7. 10.1186/s12864-021-08135-7 PMC8571842 34740333

[16] Wang X, Su F, Yu X, Geng N, Li L, Wang R, et al. RNA-seq whole transcriptome analysis of bovine mammary epithelial cells in response to intracellular *Staphylococcus aureus*. Front Vet Sci. 2020;7:642. 10.3389/fvets.2020.00642. 10.3389/fvets.2020.00642 PMC7793973 33426011

[17] Cheng Z, Buggiotti L, Salavati M, Marchitelli C, Palma-Vera S, Wylie A (2021). Global transcriptomic profiles of circulating leucocytes in early lactation cows with clinical or subclinical mastitis. Mol Biol Rep.

[18] Bakhtiarizadeh MR, Mirzaei S, Norouzi M, Sheybani N, Vafaei Sadi MS. Identification of gene modules and hub genes involved in mastitis development using a systems biology approach. Front Genet. 2020;11:722. 10.3389/fgene.2020.00722. 10.3389/fgene.2020.00722 PMC7371005 32754201

[19] Welderufael BG, Løvendahl P, de Koning DJ, Janss LLG, Fikse WF. Genome-wide association study for susceptibility to and recoverability from mastitis in Danish Holstein cows. Front Genet. 2018;9:141. 10.3389/fgene.2018.00141. 10.3389/fgene.2018.00141 PMC5932407 29755506

[20] Naserkheil M, Ghafouri F, Zakizadeh S, Pirany N, Manzari Z, Ghorbani S (2022). Multi-omics integration and network analysis reveal potential hub genes and genetic mechanisms regulating bovine mastitis. Curr Issues Mol Biol.

[21] NMC. Laboratory handbook on bovine mastitis. third. New Prague: National Mastitis Council, Inc.; 2017.

[22] Overbey EG, Saravia-Butler AM, Zhang Z, Rathi KS, Fogle H, da Silveira WA, et al. NASA geneLab RNA-seq consensus pipeline: standardized processing of short-read RNA-seq data. iScience. 2021;24(4):102361. 10.1016/j.isci.2021.102361. 10.1016/j.isci.2021.102361 PMC8044432 33870146

[23] Krueger F. Trim Galore: a wrapper around cutadapt and FASTQC to consistently apply adapter and quality trimming to FASTQ files, with extra functionality for RRBS data (version 0.6.5). 2019. https://github.com/FelixKrueger/TrimGalore .

[24] Ewels P, Lundin S, Max K. Data and text mining MultiQC : summarize analysis results for multiple tools and samples in a single report. Bioinformatics. 2016;32:3047–8. 10.1093/bioinformatics/btw354 PMC5039924 27312411

[25] Dobin A, Davis CA, Schlesinger F, Drenkow J, Zaleski C, Jha S (2013). STAR: ultrafast universal RNA-seq aligner. Bioinformatics.

[26] Li B, Dewey CN. RSEM : accurate transcript quantification from RNA-Seq data with or without a reference genome. BMC Bioinformatics. 2011;12:1–16. 10.1186/1471-2105-12-323 PMC3163565 21816040

[27] Robinson MD, Mccarthy DJ, Smyth GK. edgeR : a Bioconductor package for differential expression analysis of digital gene expression data. Bioinformatics. 2010;26:139–40. 10.1093/bioinformatics/btp616 PMC2796818 19910308

[28] Love MI, Huber W, Anders S. Moderated estimation of fold change and dispersion for RNA-seq data with DESeq2. Genome. 2014;15:550. 10.1186/s13059-014-0550-8. 10.1186/s13059-014-0550-8 PMC4302049 25516281

[29] Bindea G, Mlecnik B, Hackl H, Charoentong P, Tosolini M, Kirilovsky A (2009). ClueGO: a Cytoscape plug-in to decipher functionally grouped gene ontology and pathway annotation networks. Bioinformatics.

[30] Drier Y, Sheffer M, Domany E (2013). Pathway-based personalized analysis of cancer. Proc Natl Acad Sci U S A.

[31] Dixon P (2003). VEGAN, a package of R functions for community ecology. J Veg Sci.

[32] Clarke KR, Ainsworth M (1993). A method of linking multivariate community structure to environmental variables. Mar Ecol Prog Ser.

[33] Rohart F, Gautier B, Singh A, Lê Cao KA (2017). mixOmics: An R package for ‘omics feature selection and multiple data integration. PLoS Comput Biol.

[34] Nyholm L, Koziol A, Marcos S, Botnen AB, Aizpurua O, Gopalakrishnan S (2020). Holo-omics: integrated host-microbiota multi-omics for basic and applied biological research. iScience..

[35] Singh A, Shannon CP, Gautier B, Rohart F, Vacher M, Tebbutt SJ (2019). DIABLO: An integrative approach for identifying key molecular drivers from multi-omics assays. Bioinformatics.

[36] Hogue CW, Groll M. An automated method for finding molecular complexes in large protein interaction networks. BMC Bioinformatics. 2001;29:137–40. https://academic.oup.com/nar/article-lookup/doi/10.1093/nar/29.1.137 10.1186/1471-2105-4-2 PMC149346 12525261

[37] Chin C, Chen S, Wu H, Ho C, Ko M, Lin C. cytoHubba : identifying hub objects and sub- networks from complex interactome. BMC Syst Biol. 2014;8(Suppl 4):S11. 10.1186/1752-0509-8-S4-S11. 10.1186/1752-0509-8-S4-S11 PMC4290687 25521941

[38] Seo M, Lee HJ, Kim K, Caetano-Anolles K, Jeong JY, Park S (2016). Characterizing milk production related genes in holstein using RNA-seq. Asian-Australasian J Anim Sci.

[39] Ingvartsen KL, Moyes K (2013). Nutrition, immune function and health of dairy cattle. Animal.

[40] Wathes DC, Cheng Z, Chowdhury W, Fenwick MA, Fitzpatrick R, Morris DG, et al. Negative energy balance alters global gene expression and immune responses in the uterus of postpartum dairy cows. Physiol Genomics. 2009;39(1):1–13. 10.1152/physiolgenomics.00064.2009. 10.1152/physiolgenomics.00064.2009 PMC2747344 19567787

[41] Bobbo T, Ruegg PL, Stocco G, Fiore E, Gianesella M, Morgante M (2017). Associations between pathogen-specific cases of subclinical mastitis and milk yield, quality, protein composition, and cheese-making traits in dairy cows. J Dairy Sci.

[42] Pegolo S, Tessari R, Bisutti V, Vanzin A, Giannuzzi D, Gianesella M (2021). Quarter-level analyses of the associations among subclinical intramammary infection and milk quality, udder health, and cheesemaking traits in Holstein cows. J Dairy Sci.

[43] Behl JD, Verma NK, Tyagi N, Mishra P, Behl R, Joshi BK. The Major histocompatibility complex in bovines: a review. ISRN Vet Sci. 2012;2012:872710. 10.5402/2012/872710 PMC3658703 23738132

[44] Wieczorek M, Abualrous ET, Sticht J, Alvaro-Benito M, Stolzenberg S, Noé F, et al. Major histocompatibility complex ( MHC ) class i and MHC class ii proteins : conformational plasticity in antigen presentation. Front Immunol. 2017;8:292. 10.3389/fimmu.2017.00292. 10.3389/fimmu.2017.00292 PMC5355494 28367149

[45] Gabrilovich DI, Nagaraj S (2009). Myeloid-derived suppressor cells as regulators of the immune system. Nat Rev Immunol.

[46] Tur J, Vico T, Lloberas J, Zorzano A, Celada A. Macrophages and mitochondria: a critical interplay between metabolism, signaling, and the functional activity. 2017. 10.1016/bs.ai.2016.12.001 10.1016/bs.ai.2016.12.001 28215277

[47] Jha AK, Huang SCC, Sergushichev A, Lampropoulou V, Ivanova Y, Loginicheva E (2015). Network integration of parallel metabolic and transcriptional data reveals metabolic modules that regulate macrophage polarization. Immunity.

[48] Di Cara F, Andreoletti P, Trompier D, Vejux A, Bülow MH, Sellin J (2019). Peroxisomes in immune response and inflammation. Int J Mol Sci.

[49] Dedhar S (2000). Cell-substrate interactions and signaling through ILK. Curr Opin Cell Biol.

[50] Zhang H, Jiang H, Fan Y, Chen Z, Li M, Mao Y (2018). Transcriptomics and iTRAQ-proteomics analyses of bovine mammary tissue with *Streptococcus agalactiae*-Induced Mastitis. J Agric Food Chem.

[51] Hauck CR, Ohlsen K (2006). Sticky connections: Extracellular matrix protein recognition and integrin-mediated cellular invasion by *Staphylococcus aureus*. Curr Opin Microbiol.

[52] Mudaliar M, Tassi R, Thomas FC, Mcneilly TN, Weidt SK, Mclaughlin M (2016). Molecular BioSystems in an experimental model of *Streptococcus uberis*. Mol Biosyst.

[53] Ivaska J, Heino J (2000). Adhesion receptors and cell invasion: mechanisms of integrin-guided degradation of extracellular matrix. Cell Mol Life Sci.

[54] Kabelitz T, Aubry E, van Vorst K, Amon T, Fulde M. The role of *Streptococcus* spp. in bovine mastitis. Microorganisms. 2021;9(7):1497. 10.3390/microorganisms9071497. 10.3390/microorganisms9071497 PMC8305581 34361932

[55] Leonel Gonçalves J, Hwa In Lee S, de Paula Arruda E, Pedroso Galles D, Camargo Caetano V, Fernandes de Oliveira CA (2015). Biofilm-producing ability and efficiency of sanitizing agents against *Prototheca zopfii* isolates from bovine subclinical mastitis. J Dairy Sci.

[56] Rainard P, Cunha P, Martins RP, Gilbert FB, Germon P, Foucras G (2020). Type 3 immunity: a perspective for the defense of the mammary gland against infections. Vet Res.

[57] Roussel P, Cunha P, Porcherie A, Petzl W, Gilbert FB, Riollet C, et al. Investigating the contribution of IL-17A and IL-17F to the host response during *Escherichia coli* mastitis. Vet Res. 2015;46:56. 10.1186/s13567-015-0201-4. 10.1186/s13567-015-0201-4 PMC4462179 26062913

[58] Rainard P, Gitton C, Chaumeil T, Fassier T, Huau C, Riou M, et al. Host factors determine the evolution of infection with *Staphylococcus aureus* to gangrenous mastitis in goats. Vet Res. 2018;49:72. 10.1186/s13567-018-0564-4. 10.1186/s13567-018-0564-4 PMC6060506 30045763

[59] Cardoso TF, Quintanilla R, Castelló A, González-Prendes R, Amills M, Cánovas Á. Differential expression of mRNA isoforms in the skeletal muscle of pigs with distinct growth and fatness profiles. BMC Genomics. 2018;19:145. 10.1186/s12864-018-4515-2. 10.1186/s12864-018-4515-2 PMC5813380 29444639

[60] Yu JW, Lee MS. Mitochondria and the NLRP3 inflammasome: physiological and pathological relevance. Arch Pharm Res. 2016;39:1503–18. 10.1007/s12272-016-0827-4 27600432

[61] Backert S, Dubois H, Wullaert A, Lamkanfi M (2016). Inflammasome signaling and bacterial infections. Curr Top Microbiol Immunol.

[62] Moyes KM, Drackley JK, Morin DE, Bionaz M, Rodriguez-Zas SL, Everts RE, et al. Gene network and pathway analysis of bovine mammary tissue challenged with *Streptococcus uberis* reveals induction of cell proliferation and inhibition of PPAR signaling as potential mechanism for the negative relationships between immune response and lipi. BMC Genomics. 2009;10:542. 10.1186/1471-2164-10-542. 10.1186/1471-2164-10-542 PMC2784807 19925655

[63] Huma ZI, Sharma N, Kour S, Tandon S, Guttula PK, Kour S (2020). Putative biomarkers for early detection of mastitis in cattle. Anim Prod Sci.

[64] Li L, Tang W, Zhao M, Gong B, Cao M, Li J (2021). Study on the regulation mechanism of lipopolysaccharide on oxidative stress and lipid metabolism of bovine mammary epithelial cells. Physiol Res.

[65] van Altena SEC, de Klerk B, Hettinga KA, van Neerven RJJ, Boeren S, Savelkoul HFJ (2016). A proteomics-based identification of putative biomarkers for disease in bovine milk. Vet Immunol Immunopathol.

[66] Devaiah BN, Singer DS. CIITA and its dual roles in MHC gene transcription. Front Immunol. 2013;4:476. 10.3389/fimmu.2013.00476. 10.3389/fimmu.2013.00476 PMC3868913 24391648

[67] Canive M, Badia-Bringué G, Vázquez P, Garrido JM, Juste RA, Fernandez A, et al. A genome-wide association study for tolerance to paratuberculosis identifies candidate genes involved in DNA packaging, DNA damage repair, innate immunity, and pathogen persistence. Front Immunol. 2022;13:965. 10.3389/fimmu.2022.820965. 10.3389/fimmu.2022.820965 PMC9019162 35464478

[68] Guzzo N, Sartori C, Mantovani R (2018). Genetic parameters of different measures of somatic cell counts in the Rendena breed. J Dairy Sci.

[69] Pilla R, Malvisi M, Snel GGM, Schwarz D, König S, Czerny CP (2013). Differential cell count as an alternative method to diagnose dairy cow mastitis. J Dairy Sci.

[70] Televičius M, Juozaitiene V, Malašauskienė D, Antanaitis R, Rutkauskas A, Urbutis M, et al. Inline milk lactose concentration as biomarker of the health status and reproductive success in dairy cows. Agriculture. 2021;11(1):38. 10.3390/agriculture11010038.

[71] Forsbäck L, Lindmark-Månsson H, Andrén A, Svennersten-Sjaunja K (2010). Evaluation of quality changes in udder quarter milk from cows with low-to-moderate somatic cell counts. Animal.

[72] Le Roux Y, Laurent F, Moussaoui F. Polymorphonuclear proteolytic activity and milk composition change. Vet Res. 2003;34:629–45. http://www.edpsciences.org/10.1051/vetres:2003021 10.1051/vetres:2003021 14556698

[73] Norberg E, Hogeveen H, Korsgaard IR, Friggens NC, Sloth KHMN, Løvendahl P (2004). Electrical conductivity of milk: ability to predict mastitis status. J Dairy Sci.

[74] Ebrahimie E, Ebrahimi F, Ebrahimi M, Tomlinson S, Petrovski KR. A large-scale study of indicators of sub-clinical mastitis in dairy cattle by attribute weighting analysis of milk composition features : highlighting the predictive power of lactose and electrical conductivity. J Dairy Res. 2018;85:193–200. 10.1017/S0022029918000249 29785910

[75] Antanaitis R, Juozaitienė V, Jonike V, Baumgartner W, Paulauskas A. Milk lactose as a biomarker of subclinical mastitis in dairy cows. Animals. 2021;11(6):1736. 10.3390/ani11061736. 10.3390/ani11061736 PMC8230553 34200862

[76] Chen Y, Jing H, Chen M, Liang W, Yang J, Deng G, et al. Transcriptional profiling of exosomes derived from *Staphylococcus aureus* -infected bovine mammary epithelial cell line MAC-T by RNA-seq analysis. Oxid Med Cell Longev. 2021;2021:8460355. 10.1155/2021/8460355. 10.1155/2021/8460355 PMC8342165 34367468

[77] Cheng Z, Palma-Vera S, Buggiotti L, Salavati M, Becker F, Werling D, et al. Transcriptomic analysis of circulating leukocytes obtained during the recovery from clinical mastitis caused by *Escherichia coli* in Holstein dairy cows. Animals. 2022;12(16):2146.10.3390/ani12162146. 10.3390/ani12162146 PMC9404729 36009735

[78] Pan C, Yang C, Ma Y, Sheng H, Lei Z, Wang S, et al. Identification of key genes associated with early calf-hood nutrition in subcutaneous and visceral adipose tissues by co-expression analysis. Front Vet Sci. 2022;9:831129. 10.3389/fvets.2022.831129 PMC9127810 35619603

