# Supplementary figures and images for: Transcriptome-wide mapping of milk somatic cells upon subclinical mastitis infection in dairy cattle
Source: J Anim Sci Biotechnol. 2023 Jul 5;14:93. doi: 10.1186/s40104-023-00890-9 (PMC10320993; doi:10.1186/s40104-023-00890-9)

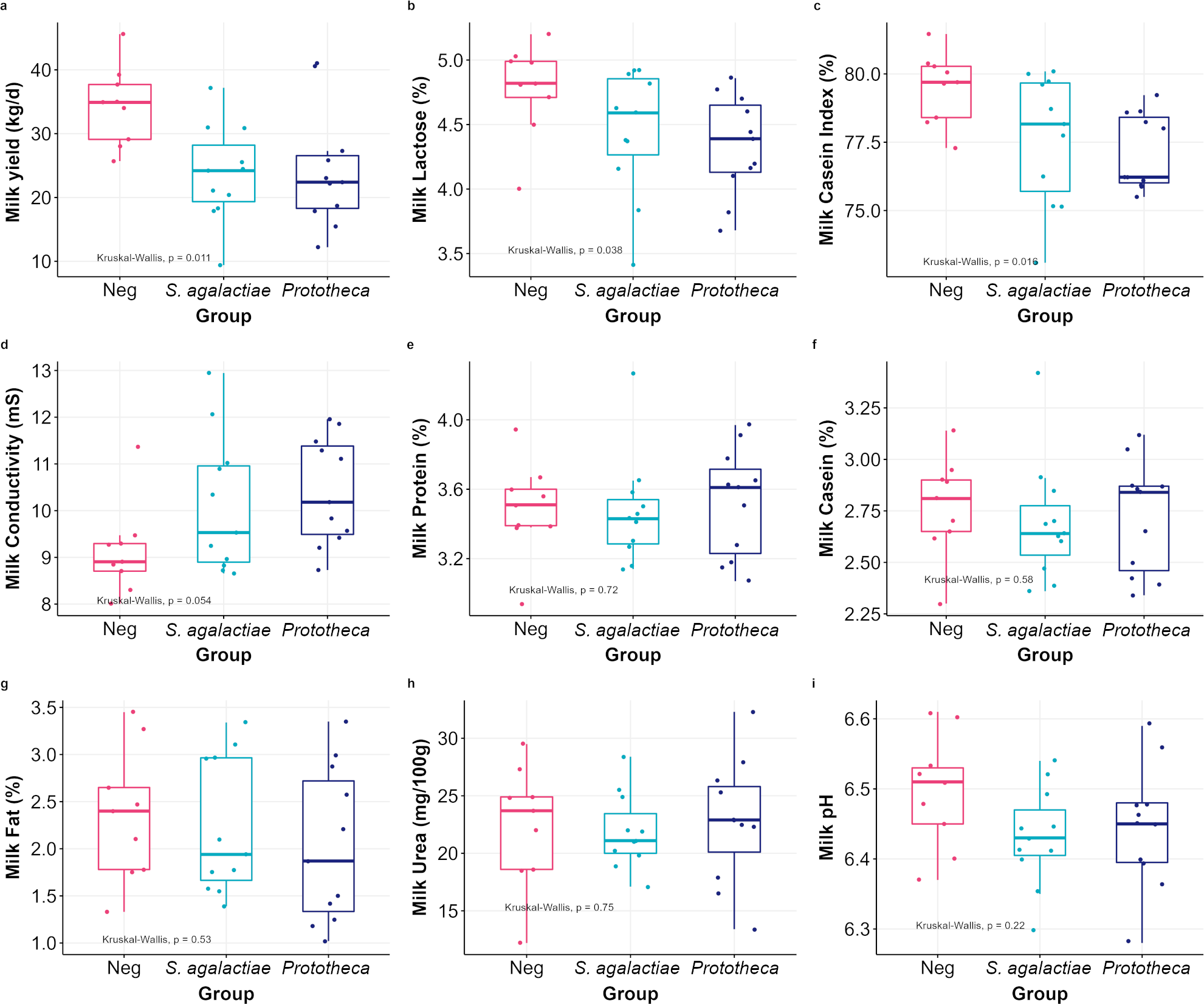

Supplement: Supplementary file 3 — Additional file 3: Fig S1. Variation of milk phenotypic traits in healthy, Prototheca’s and S. agalactiae’s infected animals. Boxplots of milk yield, milk lactose, milk casein index, milk conductivity, milk protein, milk casein, milk fat, milk urea and milk pHaccording to the three experimental groups. [file 40104_2023_890_MOESM3_ESM.tif]

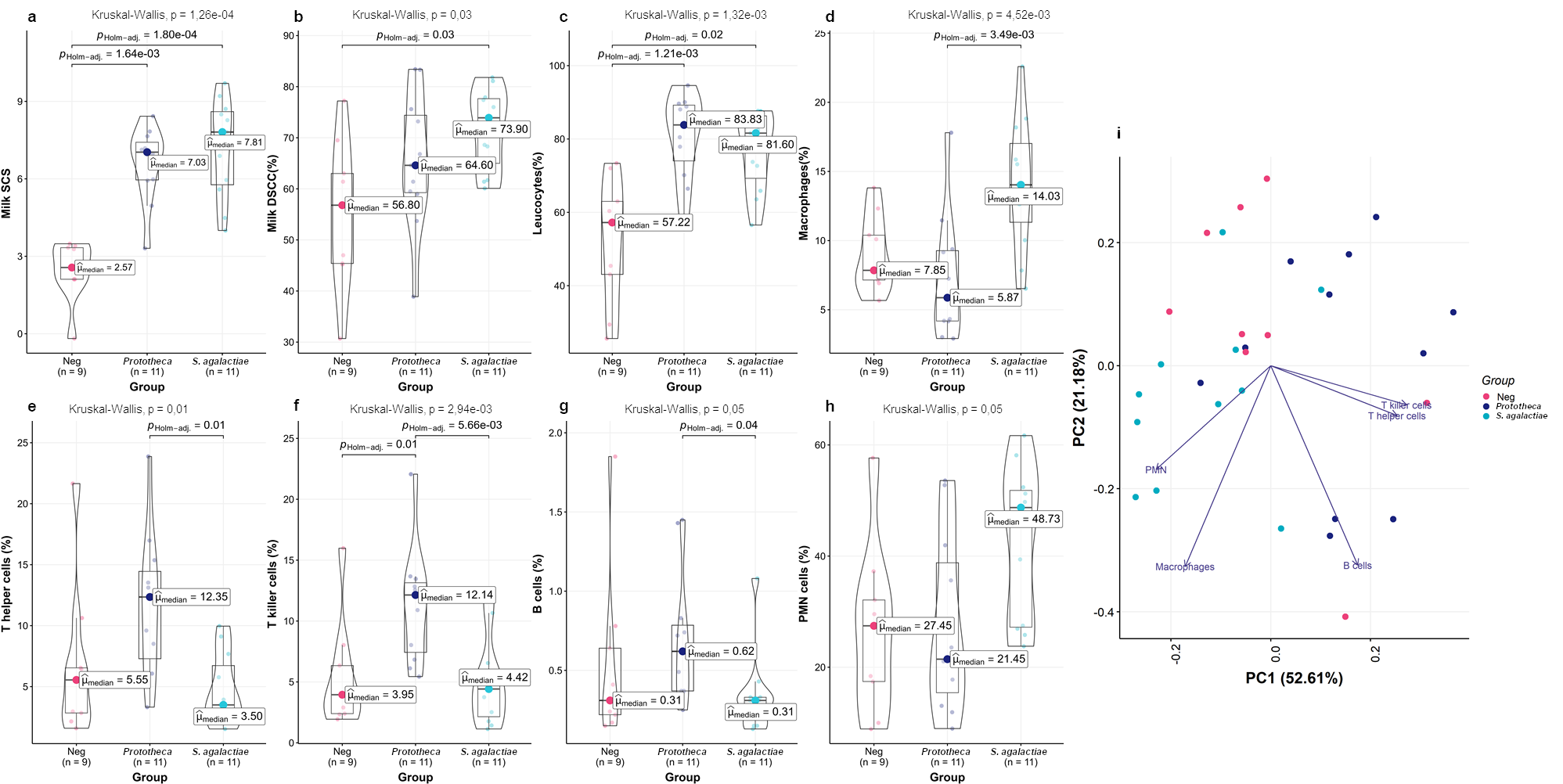

Supplement: Supplementary file 4 — Additional file 4: Fig. S2. Flow cytometry results for immune cell populations according to the three experimental groups. Violin plots of milk somatic cell score, differential somatic cell count, leucocytes, macrophages, T helper cells, T killer cells, B cellsand, PMNin healthy, Prototheca’s and S. agalactiae’s infected animals.Principal component analysis shows the samples’ separation according to the flow cytometry variables. [file 40104_2023_890_MOESM4_ESM.tif]

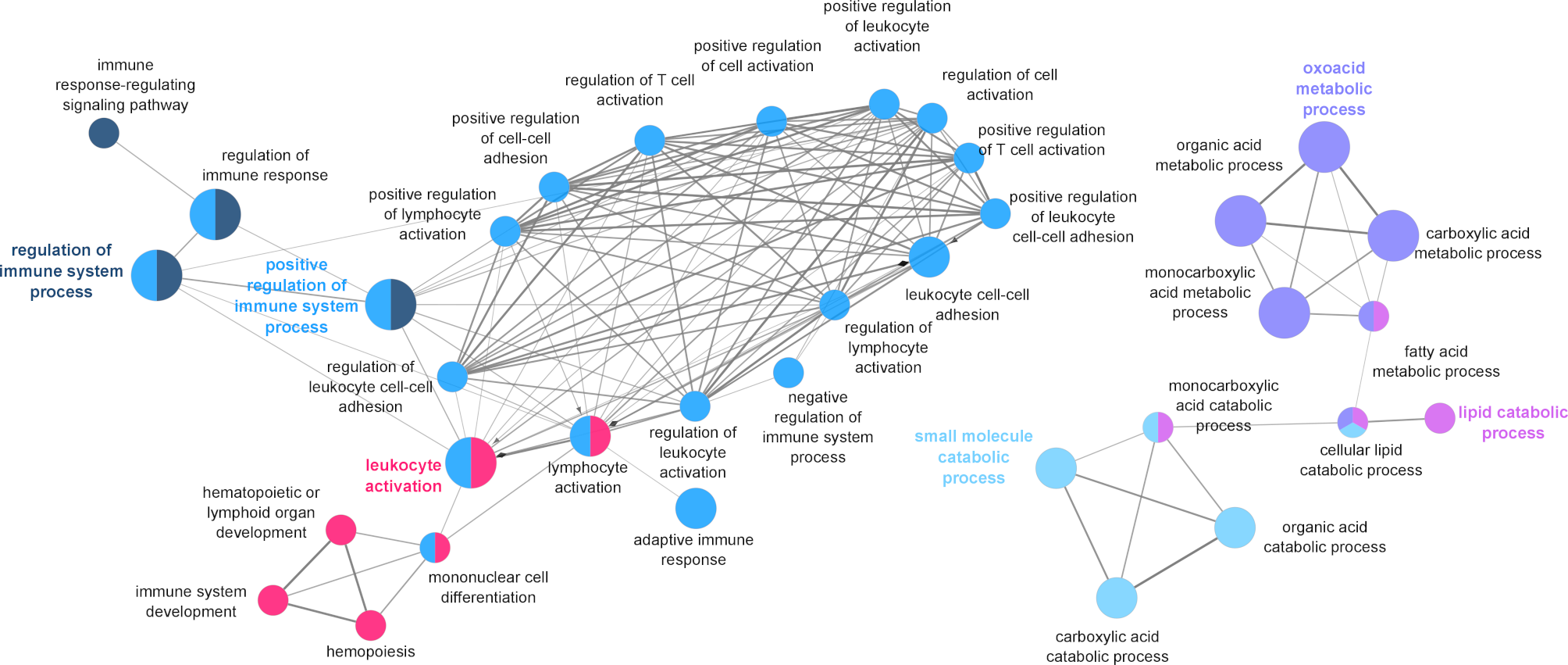

Supplement: Supplementary file 7 — Additional file 7: Fig. S3. ClueGo pathway analysis of the 681 “core mastitis response genes” commonly shared between S. agalactiae and Prototheca. [file 40104_2023_890_MOESM7_ESM.tif]

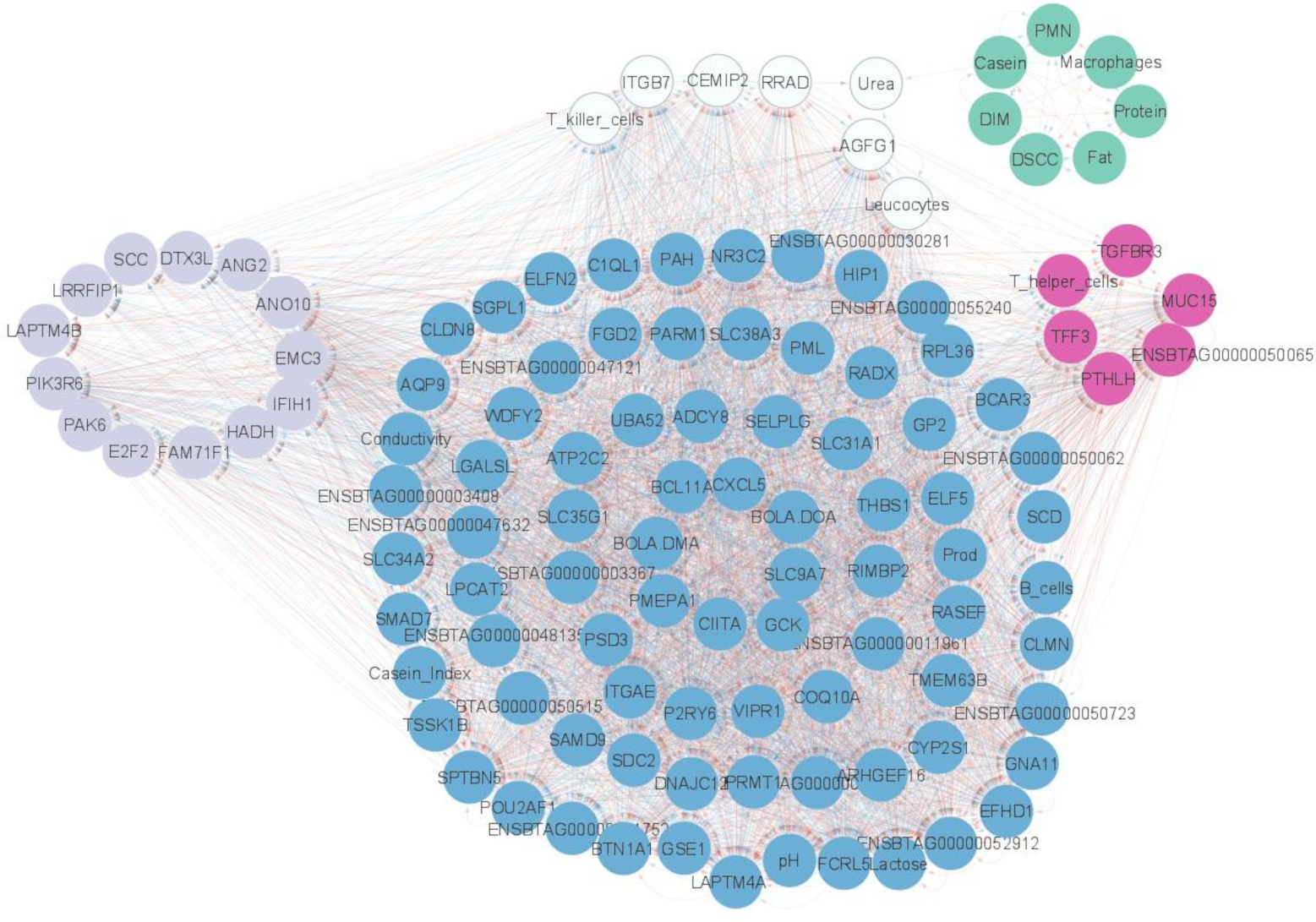

Supplement: Supplementary file 8 — Additional file 8: Fig. S4. PPI network construction and module analysis carried out with Cytoscape’s plug-in MCODE. Nodes belonging to different modules are differently colored. White nodes are variables that were not assigned to any modules. Lines represent the interaction between nodes. [file 40104_2023_890_MOESM8_ESM.tif]
